# Multiple expansions of globally uncommon SARS-CoV-2 lineages in Nigeria

**DOI:** 10.1038/s41467-022-28317-5

**Published:** 2022-02-03

**Authors:** Egon A. Ozer, Lacy M. Simons, Olubusuyi M. Adewumi, Adeola A. Fowotade, Ewean C. Omoruyi, Johnson A. Adeniji, Oluseyi A. Olayinka, Taylor J. Dean, Janet Zayas, Pavan P. Bhimalli, Michelle K. Ash, Almoustapha I. Maiga, Anou M. Somboro, Mamoudou Maiga, Adam Godzik, Jeffrey R. Schneider, João I. Mamede, Babafemi O. Taiwo, Judd F. Hultquist, Ramon Lorenzo-Redondo

**Affiliations:** 1 Division of Infectious Diseases, Department of Medicine, Northwestern University Feinberg School of Medicine, Chicago, IL USA; 2 Center for Pathogen Genomics and Microbial Evolution, Northwestern University Havey Institute for Global Health, Chicago, IL USA; 3 Department of Virology, College of Medicine, University of Ibadan, Ibadan, Nigeria; 4 Infectious Disease Institute, College of Medicine, University of Ibadan, Ibadan, Nigeria; 5 Biorepository and Clinical Virology Laboratory, College of Medicine, University College Hospital, University of Ibadan, Ibadan, Nigeria; 6 Department of Microbial Pathogens and Immunity, Rush University Medical Center, Chicago, IL USA; 7 University Clinical Research Center (UCRC), University of Sciences, Techniques et Technologies of Bamako (USTTB), Bamako, Mali; 8 School of Laboratory Medicine and Medical Sciences, University of KwaZulu-Natal, Durban, South Africa; 9 Biomedical Engineering and Preventive Medicine Department, Northwestern University, Evanston, IL USA; 10 Biosciences Division, University of California Riverside School of Medicine, Riverside, CA USA

**Keywords:** Phylogenomics, Viral infection, SARS-CoV-2, Epidemiology

## Abstract

Disparities in SARS-CoV-2 genomic surveillance have limited our understanding of the viral population dynamics and may delay identification of globally important variants. Despite being the most populated country in Africa, Nigeria has remained critically under sampled. Here, we report sequences from 378 SARS-CoV-2 isolates collected in Oyo State, Nigeria between July 2020 and August 2021. In early 2021, most isolates belonged to the Alpha “variant of concern” (VOC) or the Eta lineage. Eta outcompeted Alpha in Nigeria and across West Africa, persisting in the region even after expansion of an otherwise rare Delta sub-lineage. Spike protein from the Eta variant conferred increased infectivity and decreased neutralization by convalescent sera in vitro. Phylodynamic reconstructions suggest that Eta originated in West Africa before spreading globally and represented a VOC in early 2021. These results demonstrate a distinct distribution of SARS-CoV-2 lineages in Nigeria, and emphasize the need for improved genomic surveillance worldwide.

## Abstract (web summary)

SARS-CoV-2 genomic surveillance has been important for informing pandemic responses but many regions remain under-sampled, limiting knowledge of circulating strains. Here, the authors sequence 378 isolates from Nigeria and identify two strains that appear to be important locally though globally uncommon.

## Introduction

Almost two years after its emergence in the Hubei province of China, the continued spread of severe acute respiratory syndrome coronavirus 2 (SARS-CoV-2) across the globe has sparked a worldwide health crisis^1–3^. Despite the relatively low mutation rate of this virus, its high prevalence in the human population globally has allowed it to diversify quickly^4,5^. Identification and tracking of these mutations through whole-genome sequencing efforts have been critical to identifying routes of transmission, mapping outbreaks across communities over time, and characterizing new variants that may change the virological or clinical aspects of the disease^6–9^. For example, during the spring and summer of 2020, a mutation in the viral Spike protein, D614G, was identified in association with higher viral loads in patient upper airways^10,11^. The rapid expansion of this variant in communities across the world coupled with virological assays in vitro and in vivo, suggested that this variant is more transmissible than earlier lineages^12–15^. Indeed, this mutation is nearly fixed in the global SARS-CoV-2 population today^11^.

Continued surveillance efforts by public health entities such as the World Health Organization (WHO) and Centers for Disease Control (CDC) have designated ‘variants of concern (VOC)’ associated with increased transmissibility, increased disease severity, decreased susceptibility to therapeutic agents, and/or decreased susceptibility to antibody neutralization^16^. Lineages classified as ‘variants of interest (VOI)’ are those with genetic markers predicted to affect transmission, immune escape, therapeutic resistance, and/or diagnostic evasion^17^. These designations reflect concerns that more transmissible variants could dampen the effectiveness of current public health practices designed to limit the spread of the virus^18–20^. Some mutations may additionally impact considerations for clinical care by either altering severity of the disease or dampening the effectiveness of therapeutic monoclonal antibodies or other antiviral treatments^21^. Finally, changes to the viral Spike protein may render current vaccine formulations less efficacious and/or confer the increased capacity for re-infection^22–24^.

As the COVID-19 pandemic has progressed and evolved, designations of lineages as VOCs or VOIs has changed and has often differed between public health entities. Throughout early- and mid-2021, there were four VOCs named using the dynamic Pangolin nomenclature for SARS-CoV-2 (cov-lineages.org) and the WHO variant naming scheme: the B.1.1.7 lineage (Alpha variant) originally identified in the United Kingdom, the B.1.351 (Beta variant) originally identified in South Africa, the P.1 (Gamma variant) originally identified in Brazil, and the most recent B.1.617.2 and related AY lineages (Delta variant) initially identified in India^25–30^. As of late September 2021, as Delta has become the predominant variant worldwide, the CDC has updated its VOC classification to include only Delta^31^. Previously VOIs included the B.1.525 (Eta), B.1.526 (Iota), C.37 (Lambda), and B.1.621 (Mu) lineages, however both the WHO and CDC have recently removed all lineages from the VOI classification and have instead designated these and others as ‘variants under monitoring (VUM)’ or ‘variants being monitored (VBM)’, respectively^31^. Addressing the threats posed by variants of concern and other variants with enhanced epidemic or pandemic potential requires continued surveillance of SARS-CoV-2 genetic diversity worldwide and the ongoing assessment of the impact of novel mutations or combinations of mutations on viral fitness, transmission, antibody neutralization, therapeutics, and pathogenesis.

While many countries have developed extensive genetic surveillance and reporting systems, several regions across the globe remain critically undersampled^32^. For example, despite remarkable efforts by multiple groups and institutions^33^, several countries in Africa have reported only a handful of sequences relative to their cumulative case counts, if any^34,35^. Nigeria, the most populated country in Africa and the seventh most populated country worldwide, has only a small number of reported SARS-CoV-2 sequences available in public repositories (currently just over 1400). Through the beginning of 2021, the majority of available sequences had been generated through the African Centre of Excellence for Genomics of Infectious Disease (ACEGID), which serves a number of African countries (www.acegid.org), but more recent data has been limited. Given Nigeria’s status as an epicenter of commerce and travel in Africa, undetected expansion of a more infectious, virulent or immune-resistant variant could have major repercussions for the wider region or the entire continent. A more consistent and higher volume of sample collection and sequencing in Nigeria is required to strengthen the public health value of COVID-19 surveillance efforts.

To better understand the SARS-CoV-2 population structure in Nigeria in 2021, to add more sequences from this region to public repositories, and to perform longitudinal analyses of viral variation concurrent with the highest incidence period in the country, we performed viral whole-genome sequencing on 378 SARS-CoV-2 isolates collected from COVID-19 patients in Oyo state, Nigeria between July of 2020 and August of 2021. Oyo state’s capital city, Ibadan, is the third most populous city in Nigeria situated ~130 km away from Lagos International Airport. Oyo state is the ideal location to study both imported and locally circulating SARS-CoV-2 lineages due to its proximity to the Republic of Benin and an international airport, and its blended composition of urban and rural areas. In addition, Oyo is home to the University of Ibadan College of Medicine that provides health services for the entire region, casting a wide network for sample collection across Nigeria. Phylogenetic analysis revealed two primary lineages circulating in the region during early 2021, the B.1.1.7 or ‘Alpha’ VOC, and the B.1.525 or ‘Eta’ lineage, previously designated as a VOI. The Eta lineage outcompeted Alpha and even persisted in the region after the introduction of the Delta VOC in April of 2021. Using a pseudotyped virus system, we found that mutations in the Spike protein of Eta isolates enhanced viral entry ex vivo. Furthermore, these mutations decreased the effectiveness of antibodies from previously infected individuals to neutralize the virus, though antibodies from individuals immunized with mRNA vaccines were still effective. Interestingly, while Delta variant became dominant in Nigeria similarly to what can be observed globally, we observe that a majority of cases in Nigeria are due to a rare Delta lineage, AY.36, which constitutes over 80% of Delta cases in Nigeria, but less 0.5% of Delta cases globally. Together, these data add to SARS-CoV-2 genomic surveillance in Nigeria, identify two lineages of note that dominated the epidemic during the period of highest incidence in the region, suggest that the Eta lineage may share features with previous and current variants of concern, and identify the recent dominance of very uncommon Delta lineage. Improved and ongoing genomic surveillance in under-sampled regions across the globe will be necessary for the effective and accurate understanding of potential health risks and for their effective communication to communities worldwide.

## Results

### Specimen characteristics

**Fig. 1:**
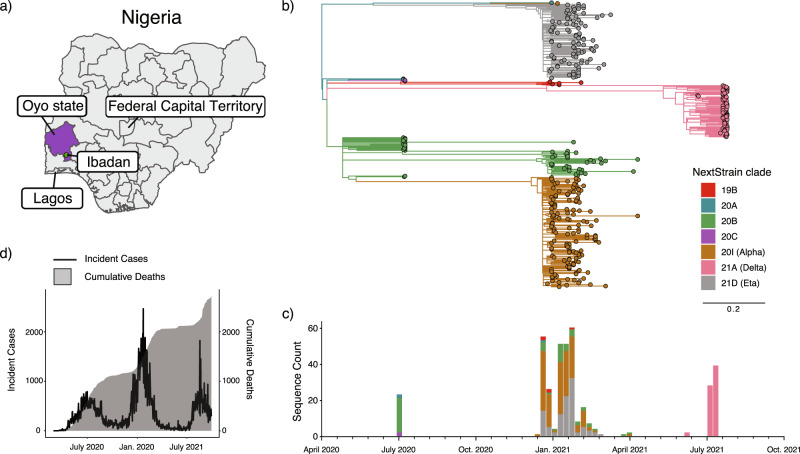
Phylogenetic analysis of SARS-CoV-2 isolates in Oyo state. **a** Geographical location of Oyo state, Nigeria is indicated (purple), where the sampling for this study was performed. **b** ML phylogenetic tree of 378 SARS-CoV-2 specimen genomes in Oyo state collected between 12 July 2020 and 3 August 2021. Clades following Nexclade nomenclature are indicated and colored. Scale indicates the number of substitutions per site and time. **c** Distribution of the different clades per time found in the Oyo dataset reported here. **d** Evolution of daily COVID-19 incidence and cumulative daily COVID-19-related deaths.

We obtained 421 specimens from individuals with COVID-19 in Oyo state, Nigeria (Fig. 1a) collected between 12 July 2020 and 3 August 2021. These specimens consisted of residual nasopharyngeal and oropharyngeal swabs that tested positive for SARS-CoV-2 by quantitative reverse transcriptase PCR (qRT-PCR) diagnostic testing in the Biorepository and Clinical Virology Laboratory at the University College Hospital, College of Medicine at the University of Ibadan. The qRT-PCR cycle threshold (Ct) values of these specimens obtained in the clinical laboratory ranged from 13.46 to 42.87 for the N target (either DaAn Gene or BGI detection kits) and 14.23 to 41.56 for the ORF1ab target (DaAn Gene kit). The average age of the patients was 42 years (range 4–86) and the sex distribution was 44% female, 37% male, and 18% unknown. RNA was extracted from each sample and the presence of viral RNA was confirmed by one-step qRT-PCR (CDC assay, RNaseP, and N1 primer set)^36^. RNA samples of sufficient quality (RNaseP control, Ct value <35) and with sufficient copies of the viral genome for sequencing (N1, Ct value <32) were reverse transcribed into complementary DNA (cDNA). The SARS-CoV-2 genome was subsequently amplified by multiplex PCR using the ARTIC protocol (primer set version 3) and subjected to deep sequencing on the Illumina platform^37,38^. The minimum threshold for base calling was 10 reads with 90% coverage required across the genome to report the whole-genome sequence. Of the 421 specimens, 43 failed to yield a satisfactory consensus sequence due to insufficient genetic material, insufficient purity after barcoding, or inadequate read coverage after sequencing; these samples were excluded from further analysis. The final 378 complete SARS-CoV-2 genomes were deposited in the public GISAID database (Supplementary Data 1) and subjected to phylogenetic analysis^39,40^.

### The lineage of concern B.1.1.7 and the lineage of interest B.1.525 dominated the epidemic in Oyo state until the arrival of the Delta lineages

We first performed phylogenetic reconstruction using maximum likelihood (ML) phylogenetic analysis of the 378 SARS-CoV-2 genomes in this study using IQ-Tree v2.0.5. The dates of sample collection were subsequently integrated to build a temporal tree using TreeTime v0.7.6 (Fig. 1b). Between January 2021 and August 2021 most of the specimens from Oyo state (89%) belonged to one of three main clusters with very strong support at their base nodes (support: >97% aLRT and >97% UFboot). We used the Pango classification scheme (PangoLearn version 9/28/2021) to identify the lineages of the major clusters. The most abundant lineage from January to April 2021 was B.1.1.7, or Alpha (147 out of 287 sequences). This lineage is associated with the Spike N501Y mutation and was first identified in the U.K. in the fall of 2020^28^. The second most common lineage during this early part of 2021 was B.1.525, or Eta (100 of 287 sequences), which expanded over time despite being in direct competition with Alpha (Fig. 1c). This lineage contains the Spike E484K, Q677H, and F888L mutations as well as three in-frame deletions shared with B.1.1.7, including Spike 69-70del and Spike 144del (cov-lineages.org). All sequences obtained between June and August 2021 belonged to the Delta variant lineages B.1.617.2 (2 sequences), AY.36 (60 sequences), and AY.4 (7 sequences). Interestingly, the most prevalent Delta lineage in this set of isolates, AY.36, is currently at a very low prevalence worldwide, represented by just under 0.5% of the Delta sequences as of September 2021 (outbreak.info). The remaining sequences all belonged to other, previously common B.1 lineages, except for three sequences from lineage A and 2 from A.27. Viruses from lineage A and derivates spread throughout the world early in the pandemic, but are now relatively rare globally, accounting for <0.5% of isolates sequenced and deposited in August 2021 (nextstrain.org).

To better determine if these sequences in Oyo state were representative of other parts of Nigeria, we downloaded all available sequences sourced from Nigeria in the GISAID database (https://www.gisaid.org/) as of 3 August 2021 (856 sequences, excluding those reported in this study). This included sequence data from Lagos state, Osun state, Abuja, and other sequences from Oyo state collected outside this study. We followed the same approach as above and performed ML phylogenetic reconstruction to estimate the evolutionary relationships with the other available sequences. We also performed ancestral reconstruction of the most likely sequences at internal nodes as well as at transition points between geographical locations to better examine relationships between the isolates found in other Nigerian states. This analysis confirmed a distribution of the viral populations throughout the country similar to what was observed in Oyo state, but with a higher proportion of the Eta lineage. Of all sequences collected between 1 January 2021 and 30 April 2021 (*n* = 875), we observed that 46% of the sequences belonged to the Eta lineage while 32% belonged to the Alpha lineage (Supplementary Fig. 1). After 30 April 2021 (*n* = 91), 89% of the sequences belonged to Delta lineages while only 2% of the sequences belonged to Eta lineages; no sequences from Alpha lineages were isolated. Among the Delta lineages, we confirmed the high prevalence of AY.36, indicating that this was not a local observation from Oyo state. Overall, these results suggest that the distribution of lineages observed in this study was broadly reflective of the contemporaneous distribution of lineages in Nigeria, and confirms the prevalence of the Eta and Alpha lineages coincident with the period of highest overall COVID-19 case counts in the country^41^ (Fig. 1d). These lineages were subsequently replaced by the Delta lineages coincident with the third spike in cases observed after July 2021.

### Introduction and characteristics of the Alpha, Eta, and Delta lineages in Nigeria

To better understand these data in the context of the global epidemic, we subsampled global sequences from GISAID collected through 3 August 2021 from these three main lineages found in Nigeria and repeated these analyses. First, we analyzed the Alpha sequences sampled in Nigeria in the context of globally distributed Alpha sequences and performed a ML phylogenetic reconstruction (Supplementary Fig. 2). These analyses showed that most Alpha lineage isolates in Nigeria were closely related and likely had a monophyletic origin with subsequent diversification (aLRT = 90.1; Supplementary Fig. 2). This sub-lineage included a prominent sub-cluster with an additional, common mutation (aLRT = 85.1; Supplementary Fig. 2). This result suggests either one successful introduction of Alpha in Nigeria, or multiple, closely linked introductions compatible with the observed founder effect. To analyze this further, we performed Bayesian inference of all Alpha sequences available from Nigeria alongside a smaller set of subsampled Alpha sequences based on time and sequence diversity from across the globe (Supplementary Fig. 3). These analyses confirmed strong statistical support for a monophyletic origin of almost all of the Alpha sequences from this study with the most likely origin being from the United States. Using this approach, we also estimated the time to the most recent common ancestor (TMRCA) for the Alpha sub-lineage in Nigeria to be around 22 October 2020 [95% Highest Posterior Density (HPD) interval: 12 October–30 October 2020].

**Fig. 2:**
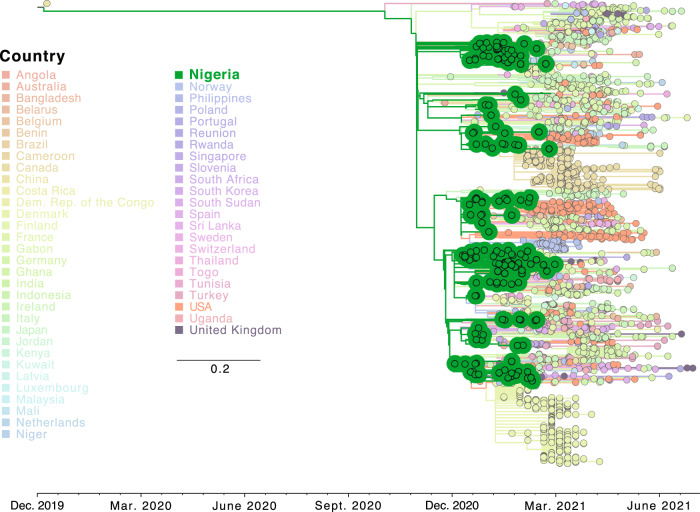
Phylogenetic analysis of the entire B.1.525 lineage. ML phylogenetic temporal reconstruction of full genome sequences from all full genome B.1.525 sequences from this study and from GISAID as of 3 August 2021. Clades corresponding to Nigerian sequences are indicated. Branches and tips are colored by country. Scale indicates number of substitutions per site and time.

The second most prevalent lineage observed in our dataset, Eta, was not as highly prevalent on a global scale as Alpha, but was previously considered a VOI and has been defined as a lineage of international significance^42^. When first detected in February of 2021 as part of our group’s ongoing genomic surveillance efforts, only 159 similar sequences were available in GISAID, a plurality (25%) of which originated in Nigeria^42^. Our continuing sampling during the first months of 2021 provided sufficient sequences for lineage designation and confirmed the high prevalence of this lineage in Nigeria compared with many other places around the globe. Lower prevalence enabled us to perform a ML analysis of our sequences with all Eta lineage sequences available in GISAID as of 3 August 2021 (*n* = 2329, not including the sequences from this study). We observed strong statistical support for a monophyletic clade of the Eta lineage in Nigeria close to the origin point, suggestive of a single introduction followed by rapid expansion soon after the initial appearance of this lineage (Fig. 2). Using a Bayesian approach, we next analyzed all Eta isolates from Oyo state relative to a subsample based on time and diversity of all Eta sequences in GISAID (Supplementary Fig. 4). The estimated TMRCA for Eta in Nigeria was 16 October 2020 [95% HPD interval: 21 September–8 November 2020], similar to the TMRCA estimated for the Alpha lineage. Together, these analyses suggest that the Eta lineage may have originated from or expanded principally in Nigeria before spreading in West Africa and globally.

**Fig. 3:**
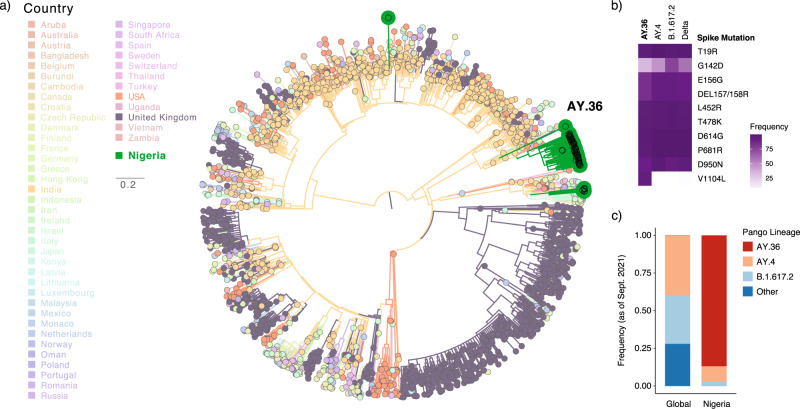
Analysis of Oyo state Delta sequences compared to global Delta sequences. **a** ML phylogenetic temporal reconstruction of full genome Delta sequences obtained in this study and temporally sampled Delta global sequences from GISAID as of 3 August 2021. Branches and tips are colored by country. The clade corresponding to the AY.36 lineage is indicated. **b** Spike protein mutation prevalence across all Delta lineages and the 2 most frequent lineages (AY.4 and B.1.617.2) compared to AY.36 lineage. **c** Prevalence of the most frequent Delta lineages globally compared to the distribution in Nigeria where AY.36 lineage was the most prevalent. AY.36 was represented in the global distribution (only 0.2% of global sequences) for comparison purposes. Data for **b** and **c** was obtained from (https://outbreak.info/)^84^.

Nearly all isolates analyzed in this study after June of 2021 belonged to one of the Delta lineages. The Delta variant has become predominant in most countries across the globe, including Nigeria. We performed a ML analysis of the Delta sequences from this study with a temporal subsample of global Delta sequences and found that a majority of sequences appeared to be monophyletic with very strong statistical support (Fig. 3). Indeed, 84% of Delta isolates in this study were classified as lineage AY.36, an uncommon Delta lineage that as of 3 August 2021 constituted <0.5% of global Delta sequences. Due to the rarity of AY.36, this analysis strongly suggests that most of the Delta variant sequences observed in Nigeria derived from a single successful introduction of AY.36 and its subsequent expansion in the country (Supplementary Fig. 5). The estimated TMRCA for AY.36 sequences in Nigeria was inferred to be 25 April 2021 [95% HPD interval: 1 April 2021–13 May 2021].

Taken together, these analyses are consistent with a single introduction or multiple, closely linked introductions of both the Alpha and Eta lineages into Nigeria in the fall of 2020. These introductions were followed by the expansion of both populations to become the predominant lineages in the country, with a wider expansion of Eta by the end of the spring of 2021. The epidemic in Nigeria has been subsequently dominated by an uncommon Delta lineage (AY.36) introduced in late April 2021, with a remnant presence of the Eta variant.

### Comparison of the SARS-CoV-2 population structure in Nigeria to other global regions

**Fig. 4:**
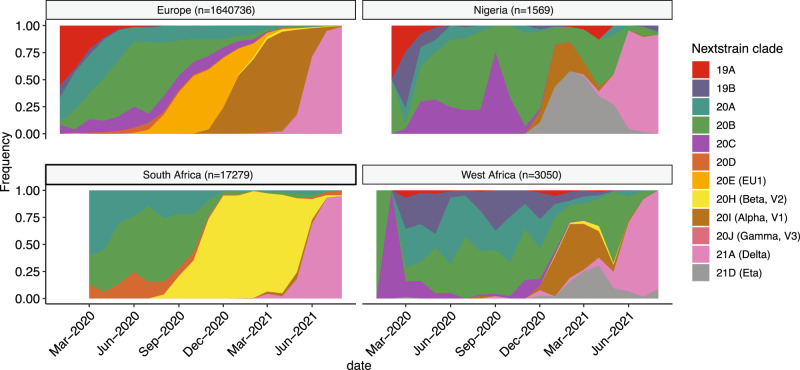
Temporal SARS-CoV-2 clade distribution in Nigeria and West Africa. Monthly clade frequency distribution using Nextclade nomenclature for clades with a frequency >1% in both Nigeria and West Africa compared to the main clades observed in Europe and South Africa. All GISAID sequences deposited up to 30 September 2021 were used to examine the period of Delta dominance. The number of sequences used per geographical location is indicated. Nextclade nomenclature was used due to its clarity to represent major clades.

The previous results suggested that the epidemic in Nigeria in early 2021 was defined by the concurrent rise of two competing variants of note, the Alpha VOC and the Eta VOI, followed by the rise of an uncommon Delta variant, AY.36. We next compared the temporal distribution of variants in Nigeria over the course of the pandemic to the rest of West Africa (excluding Nigeria), South Africa (the most widely sampled African country), and Europe (as an external and widely sampled region) (Fig. 4). This analysis shows a temporal and proportional distribution of lineages in Nigeria that is very similar to the rest of West Africa, but distinct from either Europe or South Africa. This is particularly notable in early 2021, where Alpha becomes predominant in Europe, the Beta variant becomes predominant in South Africa, but the pandemic in West Africa is defined by a competition between Alpha and Eta. These data further showed that the Eta variant appeared to outcompete Alpha in both Nigeria and West Africa as a whole before the emergence and introduction of the Delta lineages. Indeed, the Eta variant continues to circulate in West Africa as a minority of isolates in September of 2021 (https://www.gisaid.org/), even while Delta has nearly completely replaced other SARS-CoV-2 variants in other regions of the world. Moreover, to further examine the resilience of this lineage, we sampled 50 isolates (from which we were able to amplify 37 viral genomes) collected between December 2020 and August 2021 from a non-neighboring West African country, Mali. During this time period 81% of the sequences from Mali were the Eta variant, including 4 out of 4 obtained in July 2021 and 2 out of 4 obtained in August 2021 (Supplementary Fig. 6).

### Limited differences in the Ct values of the diagnostic PCR tests by lineage

The Alpha and Delta lineages have been previously associated with increased viral loads in the upper airways of patients relative to other SARS-CoV-2 lineages^43^. To assess this in our study, we compared the real-time PCR cycle threshold (Ct) values for the N1 probe measured for each isolate at the time of diagnosis as a proxy for viral load. Using a linear model to control for patient age and sex, we compared the Ct value for all 378 diagnostic specimens batched by lineage (Supplementary Fig. 7). We did not observe any statistically significant differences between the three main variants (Alpha, Eta, and Delta lineages), though both Alpha and Delta had significantly lower Ct values than 20B as previously reported^43^. Although a larger dataset that controls for a higher number of confounders will be needed to robustly test for differences, this result suggests there are comparable average viral loads in the upper respiratory tract between these three lineages at the time of patient diagnosis.

### Spike protein mutations in the B.1.525 lineage promote cell entry

Given that the expansion of Eta lineage in Nigeria occurred alongside the expansion of the Alpha VOC, we sought to characterize the phenotypic impact of the Eta Spike (S) mutations on viral infectivity. Eta lineage isolates are characterized by several nonsynonymous mutations in S at positions Q52R, A67V, E484K, D614G, Q677H, and F888L, as well as by two in-frame deletions at positions 69–70 and 144. Notably, both deletions are also found in the Alpha lineage and have been previously linked with enhanced infectivity and transmission^44^, while the E484K mutation has arisen independently in multiple lineages and has been associated with the potential for immune evasion^45^.

**Fig. 5:**
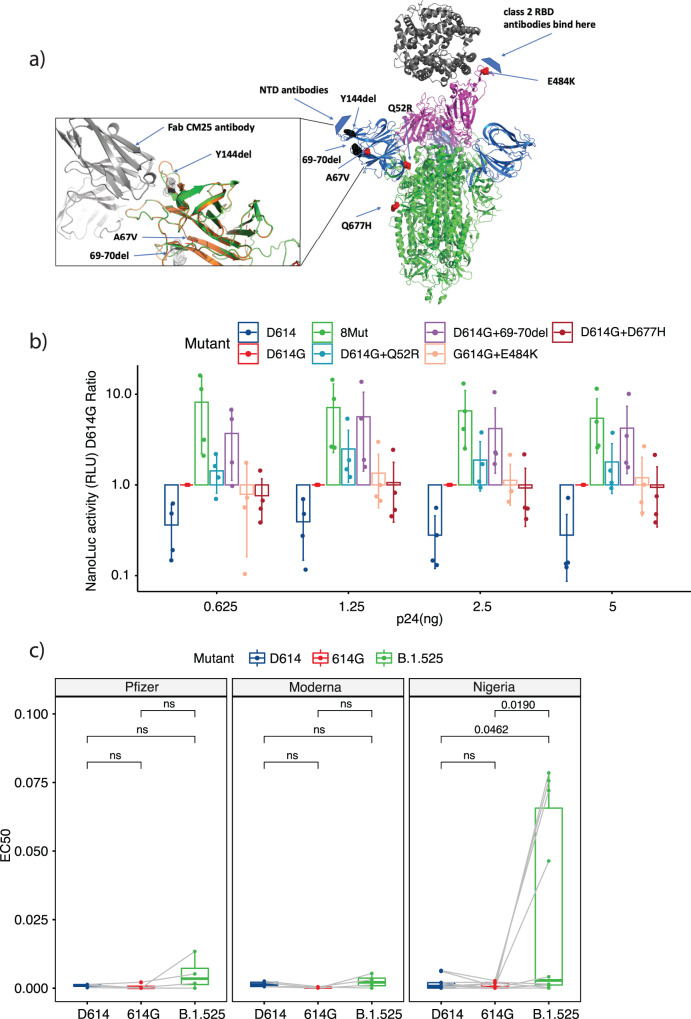
Analysis of B.1.525 Spike mutations on cellular entry and antibody neutralization. **a** Overall structure of the SARS-CoV-2 spike protein trimer with N-terminal domains (NTD) in blue, receptor-binding domains (RBD) in magenta, and ACE2 receptor in dark gray, based on the set of PDB coordinates 7a94^85^ showing the spike protein bound to one ACE2 molecule. Mutated residues in the B.1.525 spike are shown in chain A as red spheres, deleted residues as black spheres. A model of the B.1.525 NTD in the inset is shown interacting with the Fab C25 antibody (PDB 7m8j)^81^. The unmodified chain on the wild-type NTD is shown in orange. A significant conformational change in the N3 loop results in a significant drop in the (estimated) binding energy to this antibody, from −10.8 kcal/mol to −1.3. **b** Nanoluc activity measured in relative light units (RLU) ratio between each of the mutants tested and the D614G mutant. D614G was used to calculate the ratios due to its predominance in the population before the appearance of the B.1.1.7 and B.1.525 lineages. Values are shown for each of the dilutions used after p24 concentration normalization. Dots represent the value of each of the replicates performed per mutant (*n* = 4). Bars represent the mean and lines represent the standard deviation of the replicates. **c** Neutralization EC_50_ comparison between the different Spikes tested in the presence of sera from vaccinated (Pfizer [*n* = 4] or Moderna [*n* = 4] groups) or naturally-infected (Nigeria group [*n* = 9]) individuals. EC_50_ values were estimated using a four-parameter log-logistic function with the plasma dilution factors. Statistically significant FDR values are indicated for comparisons within the fitted linear mixed-effects model (ns indicates non-significant FDR using 0.05 as the cut-off). Tukey’s box and whisker plots were used; box limits: interquartile range (IQR); middle line: median; vertical lines: data range (1st quartile − 1.5 IQR; 3rd quartile + 1.5 IQR). Dots represent each serum value per mutant tested and values from the same serum for the different mutants are connected by gray lines.

Mapping the Eta lineage-defining mutations onto available Spike protein structures demonstrated an accumulation of changes in the N-terminal domain (NTD) of the protein. To better understand the impact of these mutations, we used structural modeling to examine the predicted changes in Spike protein structure in the presence and absence of binding to both ACE2 as well as NTD-directed antibody (Fig. 5a). As suggested by studies in other lineages, the E484K mutation in the receptor-binding domain (RBD) modifies a main epitope for class 2 antibodies. In addition, several other mutations in the Eta lineage are immediately adjacent to another antigenic “supersite” located in the NTD^46–48^, independent of the RBD. Deletion of the residue Y144 in particular directly impacts the central ‘N3 loop’ in the supersite while the 69–70 deletion and the A67V mutation further shift this loop. The Q52R mutation is also located in the NTD, but further from the identified antigenic region. Q677H is close to the furin cleavage site, potentially affecting the dynamics of Spike cleavage, a process required for cell entry^49^. Q677H is also seen in minor variants of Alpha and Delta, including a fast-growing AY.34 subvariant, and is currently considered a mutation of interest for increased surveillance. Finally, the F888L mutation is located in the CD1 (connecting domain 1) region of the S2 spike subunit, and there is increasing evidence that mutations in the S2 region may act synergistically with furin region mutations to increase the efficiency of the fusion process.

To test the impact of these mutations on viral entry, we generated a panel of HIV-1 luciferase reporter viruses pseudotyped with SARS-CoV-2 Spike containing the selected mutations. Given that the Spike D614G mutation is nearly fixed on the global scale, this construct was used as a baseline. On this background, each of the other eight mutations in B.1.525 were introduced both individually (with the exception of A67V that was initially not considered a lineage-defining mutation and the 144 deletion/F888L mutation that we were not able to generate) as well as cumulatively to generate an Eta lineage consensus Spike. The ancestral SARS-CoV-2 Spike D614 was also included as a control. Viral input was normalized by the concentration of the HIV p24 protein and used to challenge HeLa cells overexpressing ACE2 in step-dilution MOI in technical duplicates across two independent experiments. Successful delivery of the reporter gene was monitored by luciferase activity in the cell lysate 48 h after inoculation (Fig. 5b and Supplementary Fig. 8a). As previously reported^10–15^, the D614G mutation significantly enhanced the delivery of the reporter over the original Spike containing D614. Two mutations enhanced reporter activity even further in conjunction with the D614G mutation: the 69-70 deletion (FDR < 0.001) and the Q52R point mutation (FDR = 0.0137). Moreover, the combination of mutations contained in the Eta consensus Spike demonstrated the highest amount of viral entry relative to each tested mutation indicating an additive effect of these mutations. A western blot of virus-like particles used for cell challenge demonstrated that all generated mutants incorporated Spike protein properly into viral particles (Supplementary Fig. 8b). Together, these results suggest that the Spike mutations in the Eta lineage enhance cell entry in an in vitro system, consistent with the phenotypic impact of Spike mutations in other lineages of concern^50,51^.

### Spike protein mutations in the Eta lineage reduce antibody neutralization after natural infection

One of the biggest concerns for any new SARS-CoV-2 variant is the potential impact of accumulated Spike mutations on antibody-mediated neutralization after vaccination or natural infection. To examine the susceptibility of Eta lineage virus Spike proteins to neutralizing antibodies, HIV-1 luciferase reporter viruses were pseudotyped with a subset of the mutations tested above: the original D614 Spike protein, Spike protein containing the D614G mutation, or Spike protein containing the eight mutations that characterize the Eta lineage. Viruses were normalized by p24 concentration and incubated with sera isolated from individuals inoculated with two doses of the BNT162b2 vaccine (Pfizer, *n* = 4), individuals inoculated with two doses of the mRNA-1273 vaccine (Moderna, *n* = 4), or convalescent individuals recovered from COVID-19 infection (*n* = 9) (Supplemental Table 1). Serum from vaccinated individuals was collected roughly two weeks after the second vaccine dose had been administered. Convalescent serum had been collected from residents of Oyo state, Nigeria prior to the emergence of the Eta lineage (from 18 May 2020 to 27 June 2020) at least 5 days, but no later than 5 weeks, after diagnosis with SARS-CoV-2 infection. Antibodies against Spike RBD and NTD were quantified by ELISA in all serum specimens to confirm the presence of an immune response prior to neutralization testing (Supplementary Fig. 8c). On average, serum from vaccinated individuals carried higher antibody titers against both Spike RBD and NTD than convalescent serum. Concentrations of Spike RBD and NTD antibodies in all individuals showed a strong correlation with each other (Spearman rho = 0.93; Supplementary Fig. 8d). After incubation in different dilutions of serum, pseudotyped viruses expressing each Spike mutation were used to challenge HeLa-ACE2 cells with infection measured by luciferase activity in cell lysates after 48 h. The half-maximal effective concentration (EC_50_) was subsequently extrapolated from each dilution curve (Supplementary Fig. 9a) normalizing for differences in Spike infectivity (Supplementary Fig. 9b).

Serum from individuals fully vaccinated with either mRNA vaccine was highly protective against all Spike variants tested, with no significant differences observed between neutralization of viruses expressing the original D614 Spike, G614 Spike, or Eta Spike (Fig. 5c). Convalescent sera similarly demonstrated nearly complete neutralization of viruses carrying D614 and G614 Spike variants when compared to controls without serum treatment. However, the ability of convalescent sera to neutralize Eta Spike (median plasma dilution EC_50_ = 2.8 × 10^−3^) was significantly reduced compared to either D614 (median plasma dilution EC_50_ = 8 × 10^−4^) or G614 Spike (median plasma dilution EC_50_ = 4 × 10^−4^) (Fig. 5c). Overall Spike RBD and NTD antibody levels in each specimen correlated with the neutralization activity of D614 Spike (Supplementary Fig. 8e), suggesting that increased antibody titer following vaccination may be one correlate of improved protection. However, these results also suggest that the Eta Spike protein may confer partial escape from antibody-mediated neutralization following natural infection with a different lineage.

## Discussion

These results demonstrate the emergence and subsequent dominance of multiple, distinct SARS-CoV-2 lineages in Oyo state, Nigeria. Notably this included the coincident expansion of the B.1.1.7 Alpha ‘variant of concern’ and the B.1.525 Eta lineage in late 2020 followed by expansion of the uncommon Delta AY.36 lineage of concern by the summer of 2021. As variants with documented evidence of enhanced infectivity, transmissibility, and/or immune evasion continue to emerge and expand across the globe, updated and continued guidance is needed to ensure improved diagnostic and prevention strategies are implemented as soon as possible. The ability of Eta to outcompete Alpha in West Africa, coupled with the evidence of increased infectivity in vitro and enhanced immune evasion after natural infection, suggest that Eta may have warranted designation as a ‘variant of concern’ had its expansion potential and phenotypic characteristics been recognized earlier. More broadly, this work demonstrates the critical need for international cooperation in infectious disease surveillance in undersampled regions for the monitoring and “early-warning” detection of new pathogenic threats.

The Alpha lineage, which was first reported on 21 December 2020 in the U.K., represented a plurality of cases in Oyo state and throughout Nigeria by early January 2021. While this lineage has not been found to influence the efficacy of neutralizing antibodies produced by vaccination, it is considered a variant of concern as it expanded rapidly in multiple places across the globe, was found to be more infectious in vitro, has evidence of increased transmissibility, and can interfere with diagnostic tests probing over the 69–70 deletion in the S protein^28,52–54^. Compared to the Alpha lineage, many fewer representative cases of the Eta lineage have been reported to date, although it also expanded globally over a similar time frame. This may be due in part to undersampling of the regions where Eta was most prevalent, including Nigeria and the rest of West Africa. Indeed, phylogenetic analyses of the available sequences are consistent with this region being the source of most Eta cases observed elsewhere globally. That being said, we cannot definitively conclude that the Eta lineage originated in Nigeria due to low sampling and the observation that several sequences from Turkey root outside of the node with the highest statistical support corresponding to TMRCA for the Eta lineage in Nigeria. Importantly, the Eta lineage carries several Spike mutations that have been previously linked to transmissibility and decreased vaccine efficacy, leading to it being designated a ‘variant of interest’ by the WHO and CDC shortly after its description. Not only does the Eta variant share two Spike deletions with Alpha (69-70del and 144del), but this lineage also contains the Spike E484K mutation, which prior studies had suggested could impact immune recognition and vaccine efficacy^55–60^.

The Eta lineage showed evidence of outcompeting the Alpha lineage in Nigeria and across West Africa until the introduction and rapid expansion of the Delta lineage in late spring of 2021. Delta became predominant in the country by June of 2021 with the Eta lineage representing only a small proportion of cases, if any, by the fall. A majority of Delta isolates in our study (84%) belonged to the otherwise rare AY.36 lineage that constitutes less than 0.5% of Delta sequences globally. At the time of the study, only 11 countries (including Nigeria) out of 142 listed in GISAID had a majority of cases attributable to a lineage this rare and, of these 11 countries, only Nigeria and Israel reported more than 100 Delta lineage sequences. Besides the Spike mutations common to all Delta lineages, L452R and P681R, AY.36 also carries an additional Spike mutation, V1104L, in the C-terminus of S2. This additional mutation also appears to have arisen independently in other Delta lineages, including AY.20, AY.22, and AY.31, possibly indicating a convergent evolution process rather than founder effect in Nigeria. Additional studies of this mutation are required to determine its impact on Spike function and immunological profile.

**Fig. 6:**
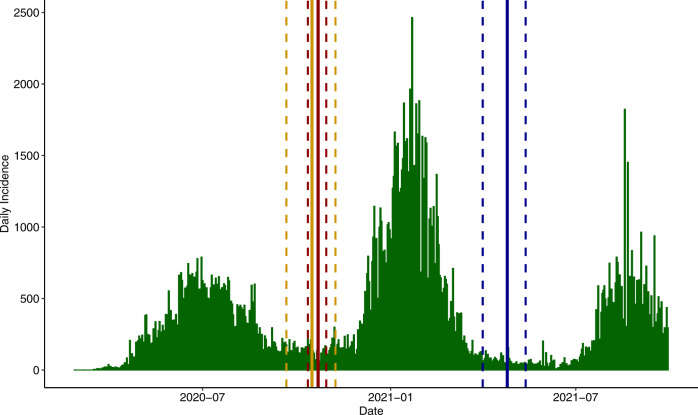
Daily SARS-CoV-2 Incidence in Nigeria. Confirmed new cases in Nigeria obtained from Johns Hopkins University Coronavirus resource center (https://coronavirus.jhu.edu/). The TMRCA (solid line) and 95% High Probability Density (HPD) (dashed lines) in Nigeria of B.1.1.7 (dark red), B.1.525 (gold), and Delta (dark blue) lineages estimated using Bayesian methods is indicated.

Comparing the estimated TMRCA for the Alpha, Eta, and Delta lineages with the daily incidence reported by the Nigerian Centers for Disease Control (Fig. 6), we note that the possible introduction or appearance of these lineages coincides with some of the lowest daily incidence numbers since the beginning of the pandemic. This suggests that these lineages may have benefited from founder effects that boosted overall prevalence of each as case numbers rose. Less than a month prior to the estimated TMRCA for Alpha and Eta lineages, Nigeria reopened international airports to regular air traffic (5 September 2020). This step was accompanied by strong measures to prevent SARS-CoV-2 introduction to the country, including requiring a negative qRT-PCR test within 96 h of boarding an in-bound flight, screening for COVID-19 symptoms prior to boarding the flight, self-quarantine for 7 days after arrival in Nigeria, and a mandatory second qRT-PCR test on day 7 of arrival. Despite these rigorous public health measures, these data are consistent with a model wherein these lineages were seeded from international travel followed by local expansion. Indeed, a similar pattern is observed with the Delta variant, which also entered the country at a time of low incidence (Fig. 6). This would be consistent with the near monophyletic origins observed for each of these lineages in Nigeria and may explain the emergence of two different otherwise rare lineages in the country.

While founder effects may have contributed to the predominance of these lineages in Nigeria following their introduction during a nadir in case counts, cell culture entry analyses conducted using Spike-pseudotyped virus particles suggest that the Spike proteins of Alpha, Delta, and now Eta also enhance viral entry. Previous studies have described the incremental improvement in SARS-CoV-2 cell entry through Spike protein mutations in lineages that subsequently expanded internationally. The D614G mutation in Spike is now nearly fixed across the global population and defines the predominant B.1 lineage from which the above lineages derive. This mutation was found to improve viral entry into airway epithelial cells and confer both increased transmissibility and increased fitness^10–15^. Subsequently, multiple further variations in Spike, including the 69-70 deletion in the Alpha and Eta lineages, have been described that improve viral entry even further over the G614 background^27,43,45,51,53,59,61^. The 69-70 deletion, as well as Q52R to a lesser degree, significantly increased Spike mediated cell entry in our assays despite being located far from the ACE2 binding site (Fig. 5a, b). A deeper examination of this region is needed to understand possible effects of these mutations on receptor binding or the mechanics of viral entry.

In addition to increased efficiency of viral entry, we also observed decreased antibody neutralization of viruses carrying Eta Spike proteins in convalescent serum from individuals living in Oyo state who had previously had COVID-19 infections. Conversely, vaccinated sera appeared to confer similar protection in concordance to other studies where they have only observed modest neutralization reductions^62^. Other SARS-CoV-2 lineages have also been shown to have increased potential for immune evasion in convalescent sera^60^, including the Beta lineage, which shares a Spike E484K mutation with Eta^30^. The E484K mutation has been shown to arise spontaneously in viruses passaged in convalescent serum^63^, further supporting its role in potentially protecting the virus from antibody responses against other non-variant Spike proteins^64,65^. Moreover, most of the S gene mutations acquired by Eta are located in the NTD of the Spike protein, possibly affecting a known antigenic site^66,67^. This observation suggests likely additional effects of this set of mutations on natural immunity, specifically on antibodies directed at the NTD of the protein^68^. As suggested by our neutralization data, these mutations could confer selective advantage against previously developed natural immunity that could in part explain the selective advantage of Eta in an environment with limited SARS-CoV-2 spread such as Nigeria, where selective advances might play an even more important role.

While the introduction and spread of these lineages during a period of very low incidence, together with their possible fitness advantages, likely facilitated the rise and dominance of these distinct lineages in Nigeria, it is less clear what drove the decline of case counts in this region following each successive wave. The implementation of stricter public health measures, increased vaccination rates, and improved weather conditions have all been considered to influence waning case counts in different countries at different times, but none of these factors are sufficient to explain the resolution of different waves in Nigeria. It is possible that increased public awareness and/or changes in individual behavior can account for some of the decline, but more study of behavioral trends in the region would be needed to investigate these associations.

In sum, the successive expansion and dominance of these two otherwise rare lineages in Nigeria, Eta and AY.36, suggest that distinct viral population dynamics are underlying the epidemic in West Africa. These results underline the critical importance of improving genomic surveillance efforts to better understand and monitor new variants as they arise in distinct regions of the globe. Additional sequencing and surveillance across these under-sampled regions, and elucidation of the biological and clinical significance of new variants, will be required to better understand and prevent emerging variants with increased fitness on a global scale that could threaten vulnerable health systems of West African countries that have avoided thus far the devastating effects of SARS-CoV-2 observed in other parts of the world.

## Methods

### Viral RNA extraction

Viral RNA was extracted from clinical specimens utilizing the QIAamp Viral RNA Minikit (Qiagen, cat. no. 52906). Clinical testing for SARS-CoV-2 presence was performed by quantitative reverse transcription and PCR (qRT-PCR) with the CDC 2019-nCoV RT-PCR Diagnostic Panel utilizing the N1 probe in SARS-CoV-2 and RNase P probes for sample quality control as previously described (IDT, cat. no. 10006713). All specimens that failed to amplify the RNase P housekeeping gene were excluded from this study. All specimens with an N1 probe cycle threshold (Ct) less than or equal to 35 were considered positive and included in this study. qRT-PCR was repeated in a random selection of specimens to validate Ct values obtained by the clinical diagnostic laboratory. Ct values from the N1 probes were used in all subsequent analyses. Ct values were calculated using QuantStudio Design and Analysis v1.5.1.

### cDNA synthesis and viral genome amplification

cDNA synthesis was performed with SuperScript IV First Strand Synthesis Kit (ThermoFisher, cat. no. 18091050) using 11 μl of extracted viral nucleic acids and random hexamers according to manufacturer’s specifications. Direct amplification of the viral genome cDNA was performed in multiplexed PCR reactions to generate ~400 bp amplicons tiled across the genome. The multiplex primer set, comprised of two non-overlapping primer pools, was created using Primal Scheme and provided by the Artic Network (version 3 release). PCR amplification was carried out using Q5 Hot Start HF Taq Polymerase (NEB, cat. no. M0493L) with 5 μl of cDNA in a 25 μl reaction volume. A two-step PCR program was used with an initial step of 98 °C for 30 s, then 35 cycles of 98 °C for 15 s followed by five minutes at 64 °C. Separate reactions were carried out for each primer pool and validated by agarose gel electrophoresis alongside negative controls. Each reaction set included positive and negative amplification controls and was performed in a space physically separated for pre- and post-PCR processing steps to reduce contamination. Amplicon sets for each genome were pooled prior to sequencing library preparation.

### Sequencing library preparation, Illumina sequencing, and genome assembly

Sequencing library preparation of genome amplicon pools was performed using the SeqWell plexWell 384 kit per manufacturer’s instructions. Pooled libraries of up to 96 genomes were sequenced on the Illumina MiSeq using the V2 500 cycle kit. Sequencing reads were trimmed to remove adapters and low-quality sequences using Trimmomatic v0.36. Trimmed reads were aligned to the reference genome sequence of SARS-CoV-2 (accession MN908947.3) using bwa v0.7.15. Pileups were generated from the alignment using samtools v1.9 and consensus sequence determined using iVar v1.2.2^69^ with a minimum depth of 10, a minimum base quality score of 20, and a consensus frequency threshold of 0 (i.e., majority base as the consensus).

### Phylogenetic analysis

Genome sequences were aligned using MAFFT v7.453 software^70^ and manually edited using MEGAX v10.1.8^71^. All Maximum Likelihood (ML) phylogenies were inferred with IQ-Tree v2.0.5^72^ using its ModelFinder function^73^ before each analysis to estimate the nucleotide substitution model best-fitted for each dataset by means of Bayesian information criterion (BIC). We assessed the tree topology for each phylogeny both with the Shimodaira–Hasegawa approximate likelihood-ratio test (SH-aLRT)^74^ and with ultrafast bootstrap (UFboot)^75^ with 1000 replicates each. TreeTime v0.7.6^76^ was used for the assessment of root-to-tip correlation, the estimation of time-scaled phylogenies and ancestral reconstruction of most likely sequences of internal nodes of the tree and transitions between geographical locations along branches. TreeTime was run using an autocorrelated molecular clock under a skyline coalescent tree prior. We used the sampling dates of the sequences to estimate the evolutionary rates and determine the best rooting of the tree using root-to-tip regression with least-squares method. For B.1.1.7 and Delta global ML phylogenies, due to the high number of sequences available, we performed a temporal subsampling using genome-sampler^77^ and sampling 100 sequences per week since the appearance of each lineage. For the B.1.525 global analysis all available 2329 high-quality sequences (high coverage and low N number) from GISAID were used.

Bayesian time-scaled phylogenetic analyses were performed for the phylogenies of B.1.1.7, B.1.525, and Delta lineages separately using a smaller subset (for computational time reasons) of global sequences from GISAID additionally subsampled by diversity in relation to the respective sequences collected from Nigeria using an identity threshold of 99.5% in genome-sampler. Due to the smaller number of B.1.525 sequences available, we included 5 B.1 global sequences subsampled by diversity, as well as the reference sequence (NC_045512) to help root the phylogenies. We used BEAST v2.5.2^78^ to estimate the date and location of the most recent common ancestors (MRCA) as well as to estimate the rate of evolution of the virus. BEAST priors were introduced with BEAUTI v2.5.2 including an uncorrelated relaxed molecular clock model with a lognormal distribution of the evolutionary rate, previous estimated evolutionary rates (8 × 10-4) as the prior for the mean, and a standard deviation of 0.004 after optimization with preliminary runs. We assumed a GTR substitution model with invariant sites, as the best-fitted model obtained with ModelFinder, and a Coalescence Bayesian Skyline to model the population size changes through time. The posterior evolutionary rate was estimated to be 6.3 × 10^−4^ for the B.1.1.7 inference 7.2 × 10^−4^ for the B.1.525 analysis and 6.4 × 10^−4^ for Delta. All the analyses indicated support for a relaxed clock as the average rate coefficient of variation was 0.6 for B.1.1.7, 0.5 for B.1.525, and 0.8 for Delta, which indicates a degree of rate autocorrelation among adjacent branches in the tree. Markov chain Monte Carlo (MCMC) runs of at least 100 million states with sampling every 5000 steps were computed. The convergence of MCMC chains was monitored using Tracer v.1.7.1, ensuring that the effective sample size (ESS) values were >200 for each parameter estimated.

### Cell lines

To produce HeLa cells (obtained from AIDS reagents) overexpressing Ace2, HEK-293T cells were transfected with psPAX2, pCMV-VSVG, and pRRL.sin.cPPT.SFFV/Ace2.IRES-puro.WPRE (a gift from Caroline Goujon; Addgene plasmid # 145839; http://n2t.net/addgene:145839; RRID:Addgene_145839). HeLa cells were then transduced with the lentiviral particles and puromycin (Invivogen) selected as in Mamede et al.^79^. Overexpression was validated by western blotting.

### Generation of SARS-CoV-2 pseudovirus

HEK-293T cells were transfected with a 3:2 ratio of NL4-3-nanoluc delta env plasmid (gift from Dr. Thomas Hope) and pCAGGS-Spike Sars-CoV2 plasmid (and introduced mutations) respectively. (The following reagent was produced under HHSN272201400008C and obtained through BEI Resources, NIAID, NIH: Vector pCAGGS Containing the SARS-Related Coronavirus 2, Wuhan-Hu-1 Spike Glycoprotein Receptor Binding Domain (RBD), NR-52309). Culture media was changed 16 h post transfection and viral particles were harvested at 48 h. Viral particles were concentrated using a 20% sucrose gradient with overnight centrifugation at 5600 rcf at 4 °C and then resuspended into fresh media at a 500x concentration. The concentrated Spike pseudoviruses were quantified using HIV-1 Gag p24 Quantikine ELISA Kit (R&D Systems), and the infectivity was determined using Hela-Ace2 cells and the Nano-Glo® Dual-Luciferase® Reporter Assay (Promega). Spike protein production was verified by western blot, using concentrated viral particles combined with laemmli buffer containing 2-mercaptoethanol, heated to 98° for 20 min. Sample concentration was standardized by p24 ELISA and samples were run on a 4–20% tris-glycine gel prior to being transferred to a PVDF membrane. The membrane was probed with Anti-SARS-CoV-2 Spike Protein S1/S2 (Invitrogen PA5-112048) and anti-p24 mAb (NIH-ARP 3537).

### Synthesis of B.1.525 lineage mutations

The introduction of the B.1.525 lineage mutations into pCAGS-Spike Sars-CoV2 plasmids (BEI) was performed by restriction digest of the plasmid, followed by NEBuilder HiFi DNA assembly (New England Biolabs) with either amplified by PCR products or synthesized DNA fragments (Genewiz). The full modified sequences were verified and confirmed by Sanger sequencing (Supplementary Methods).

### Antibody neutralization assay

HeLa-Ace2 cells were plated in 96-well black wall plates at 10^5^ cells per well and incubated at 37 °C, with 5% CO_2_ for 24 hours. Media was then removed, and serum was added using serial dilutions. Pseudotyped virus (pCAGGS-Spike Sars-CoV2 and mutants; NL4-3-nanoluc delta env) was added to each well, and cells were returned to the incubator. After a 48-hour incubation, reporter gene expression was determined with Nano-Glo® Dual-Luciferase® Reporter Assay (Promega) following the vendor’s protocol using a luminometer (Cytation3, Biotek). We estimated the amount of RBD-binding and NTD-binding antibodies at half-maximal effective concentration (EC_50_) for each serum sample by fitting a four-parameter log-logistic function to each dilution curve for both RBD and NTD using the drc v3.0-1 package in R v4.0.3. For this estimation, we used the luminescence values measured in relative light units (RLU) normalized both by the values obtained with the negative controls without virus and sera and the positive controls with each corresponding virus and no sera added.

### RBD- and NTD-binding by ELISA

Ni-NTA HisSorb 96-well plates (Qiagen) were coated with 50 μl of either His-tagged RBD (Acro Biosystems, SPD-S52H6, AA 306-527) or His-tagged NTD (Acro Biosystems, S1D-C52H6, AA13-303) at a concentration of 2 μg/ml overnight at 4 °C; all subsequent steps were performed at room temperature. Plates were blocked with 3% milk; plasma was diluted 1:500 in PBS for RBD ELISA and 1:1500 for NTD ELISA prior to adding 100 μl per well for 2 h. Plates were washed with PBS-T three times, followed by incubation with secondary anti-human IgG Fc HRP (Southern Biotech) at 1:4000 for 1 h. Plates were washed three additional times with PBS-T, followed by addition of TMB substrate for 10 min. The reaction was stopped with the addition of 3 M HCl; and plates were read at an OD 450. Anti-RBD antibody CR3022 (Creative Biolabs) was used to make a standard curve with 1:2 dilutions in the range of 0.5 μg/ml to 0.03 μg/ml, utilizing a 4-parameter fit to interpolate sample concentrations. Anti-NTD antibody SPD-M121 (Acro Biosystems) was used to make a standard curve with 1:2 dilutions in the range of 0.5 μg/ml to 0.03 μg/ml, utilizing a 4-parameter fit to interpolate sample concentrations.

### Structural modeling of B.1.525S protein

A model of the B.1.525 NTD domain, with the Q52R and A67V mutations and H69, V70 and Y144 deletions was built using SwissModel^80^ using a PDB coordinate of the Fab C25 complex with the NTD of SARS-CoV-2 (PDB 7m8j)^81^ as a template. The complex was minimized and binding energy was estimated using the EvoEF program, part of the EvoDesign pipeline^82,83^.

### Statistics

All statistical analyses were performed in in R v 4.0.3. Statistical analysis of cell entry and neutralization EC_50_ were performed fitting Linear Mixed-Effects Models using lme4 v1.1-27 package. For cell entry, we tested for significant differences in cell entry between mutants with increasing concentrations of psudotyped viruses including the interaction between mutant and p24 concentration in the model and accounting for experiment and plate effects. For neutralization EC_50_ differences we included in the model the different groups of sera, each mutant, as well as the interaction of both parameters, with the addition of the possible effect of within-donor sera correlation. Afterwards we performed every possible contrast within each model and used FDR to assess significance while controlling for multiple comparisons. A linear model including patient sex and age as confounders was fitted to test for differences in Ct value between lineages.

## Supplementary information

Supplementary Information

Description of Additional Supplementary Files

Supplementary Data 1

Supplementary Data 2

## Data Availability

The 378 complete SARS-CoV-2 genomes from Nigeria and the 37 from Mali were deposited in the public databases GISAID and GenBank (Accession numbers for both databases are provided in Supplementary Data 1). The publicly available data sources used for this study are as follows: Global sequences were obtained from GISAID database (https://www.gisaid.org/; Supplementary Data 2); nextstrain.org was used for clade information and analysis; cov-lineages.org was used for lineage information and analysis; epidemiological data from Nigeria was obtained from the Nigerian Centers for Disease Control (https://covid19.ncdc.gov.ng/) and Johns Hopkins Coronavirus resource center (https://coronavirus.jhu.edu/); additional information about lineage distribution and dynamics was obtained from outbreak.info, covariants.org, and coronavirus3d.org. Nigeria’s map was created using R packages rnaturalearth v0.1.0 and sp v1.4.6. Source data are provided with this paper.

## Source data

Source Data
