# Supplementary material for: Multiple expansions of globally uncommon SARS-CoV-2 lineages in Nigeria
Source: Nat Commun. 2022 Feb 3;13:688. doi: 10.1038/s41467-022-28317-5 (PMC8813984; doi:10.1038/s41467-022-28317-5)
Supplement: Supplementary file 1 — Supplementary Information [file 41467_2022_28317_MOESM1_ESM.pdf]

Supplementary Information for

Multiple expansions of globally uncommon SARS-CoV-2 lineages in Nigeria

Ozer et al.

This PDF file includes:

Supplementary Figures 1 to 9

Supplementary Tables 1 and 2

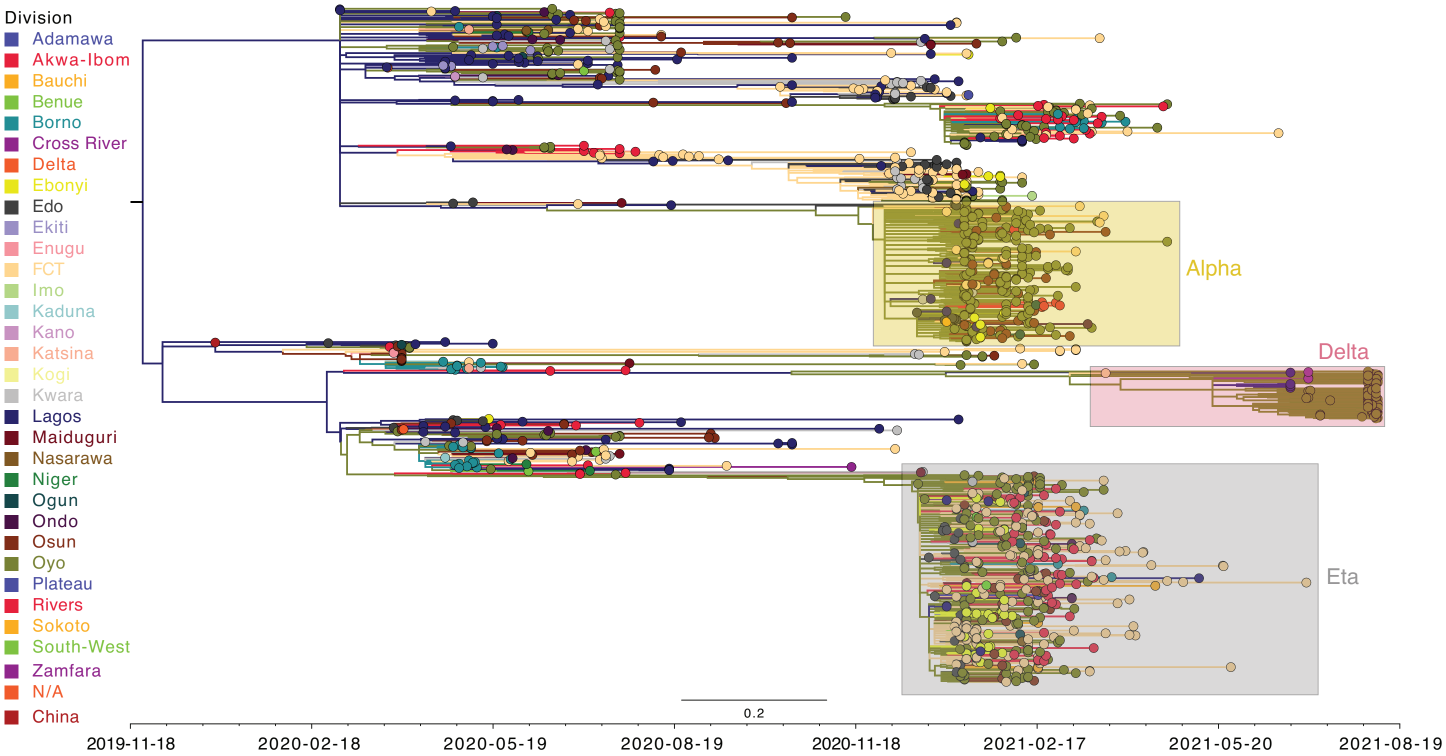

Supplementary Figure 1. Phylogenetic analysis of Oyo state isolates compared to all available Nigerian sequences. ML phylogenetic temporal reconstruction of full genome sequences from Nigeria, including the sequences from this study and all sequences available from Nigeria in GISAID as of August 3, 2021. Clades corresponding to B.1.1.7, B.1.525, and Delta lineages are indicated. Branches and tips are colored by state. Scale indicates number of substitutions per site and time.

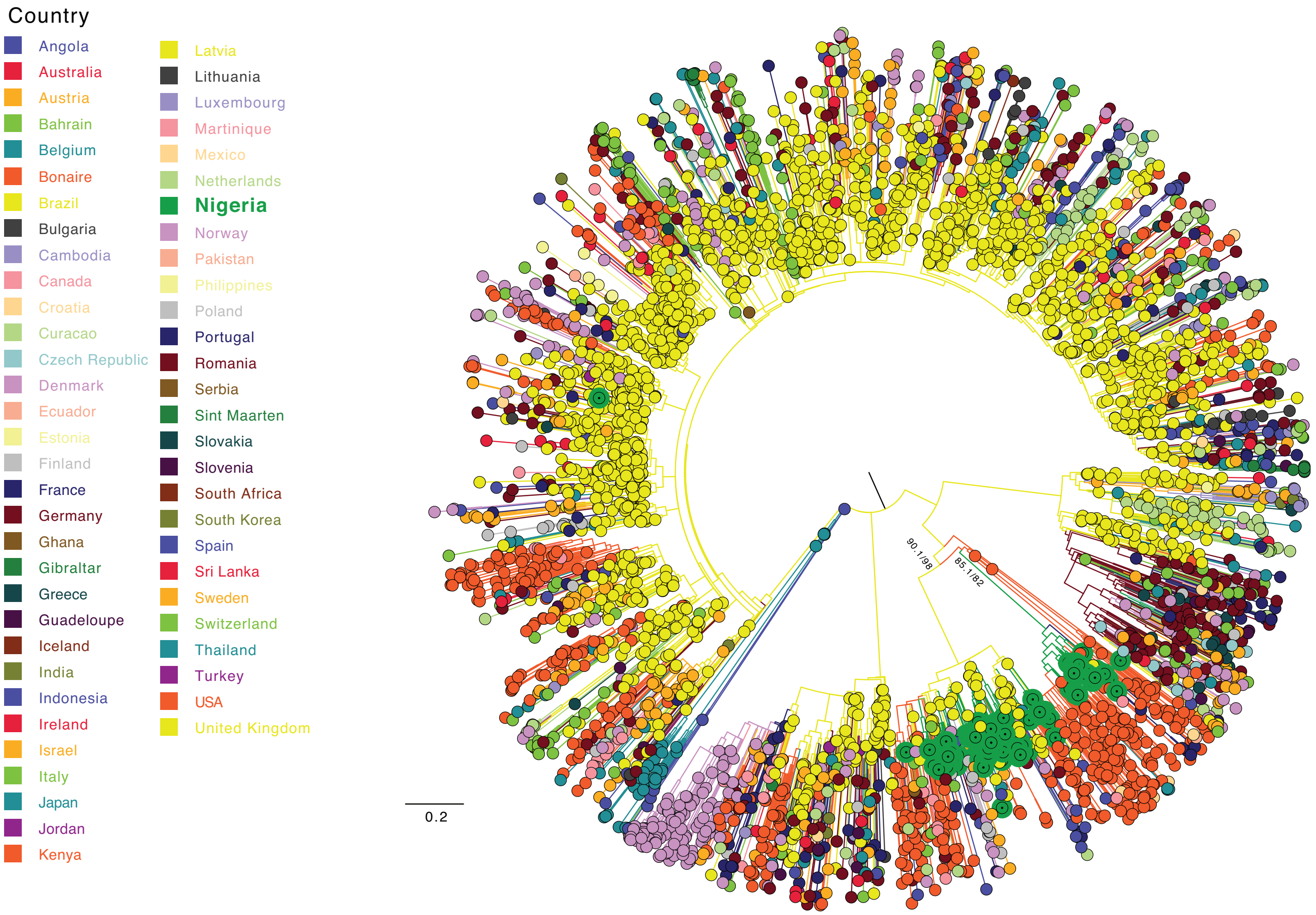

Supplementary Figure 2. Phylogenetic analysis of Oyo state B.1.1.7 sequences compared to global B.1.1.7 sequences. ML phylogenetic temporal reconstruction of full genome B.1.1.7 sequences obtained in this study and temporally sampled B.1.1.7 global sequences from GISAID as of August 3, 2021. Branches and tips are colored by country. Scale indicates number of substitutions per site and time.

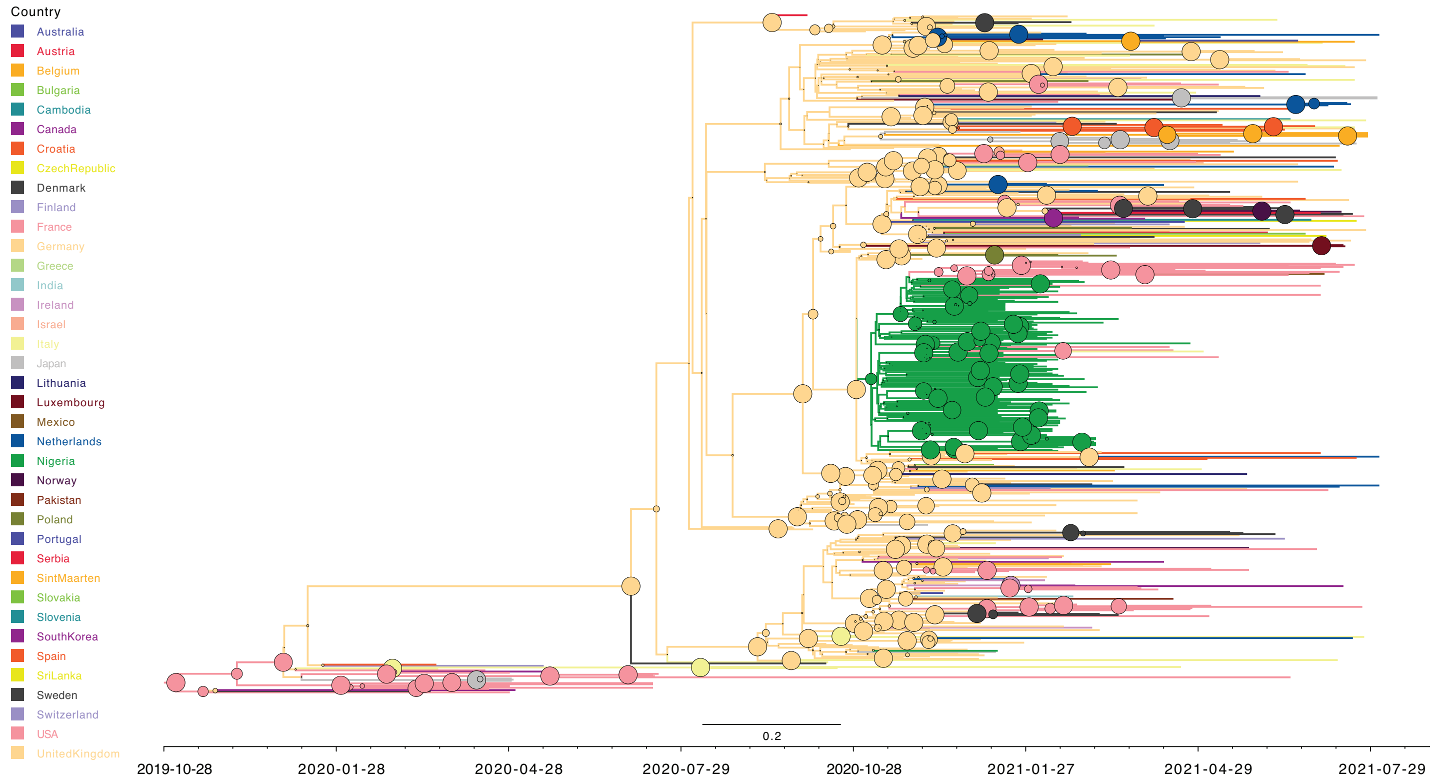

Supplementary Figure 3. Phylodynamic tree of Nigeria B.1.1.7 sequences compared to global B.1.1.7 sequences. Maximum clade credibility tree where branch colors represent the most probable geographical location of their descendent node inferred through Bayesian reconstruction of the ancestral state. All full genome B.1.1.7 sequences from Nigeria and temporally and diversity subsampled B.1.1.7 global sequences from GISAID as of August 3, 2021 were included in the analysis. The width of the node circles represents their posterior probability. Scale indicates number of substitutions per site and time.

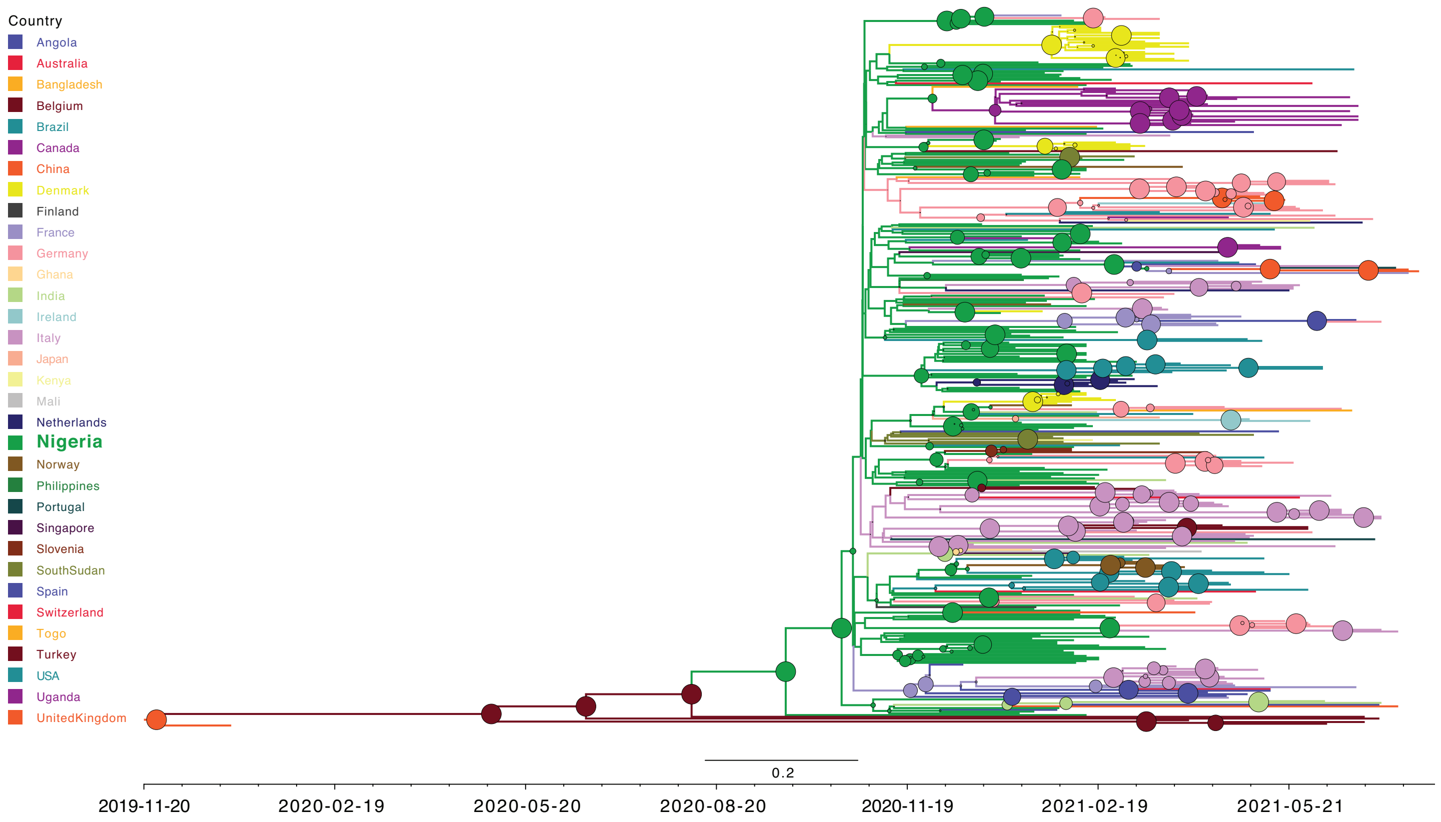

Supplementary Figure 4. Phylodynamic tree of Nigeria B.1.525 sequences compared to global B.1.525. Maximum clade credibility tree where branch colors represent the most probable geographical location of their descendent node inferred through Bayesian reconstruction of the ancestral state. All full genome B.1.525 sequences from this study and temporally and diversity subsampled from GISAID as of August 3, 2021 were included in the analysis. To root the phylogenies, the reference genome sequence NC_0455512 and 5 B.1 lineage sequences of isolates subsampled by diversity from other countries were included. The width of the node circles represents their posterior probability. Scale indicates number of substitutions per site and time.

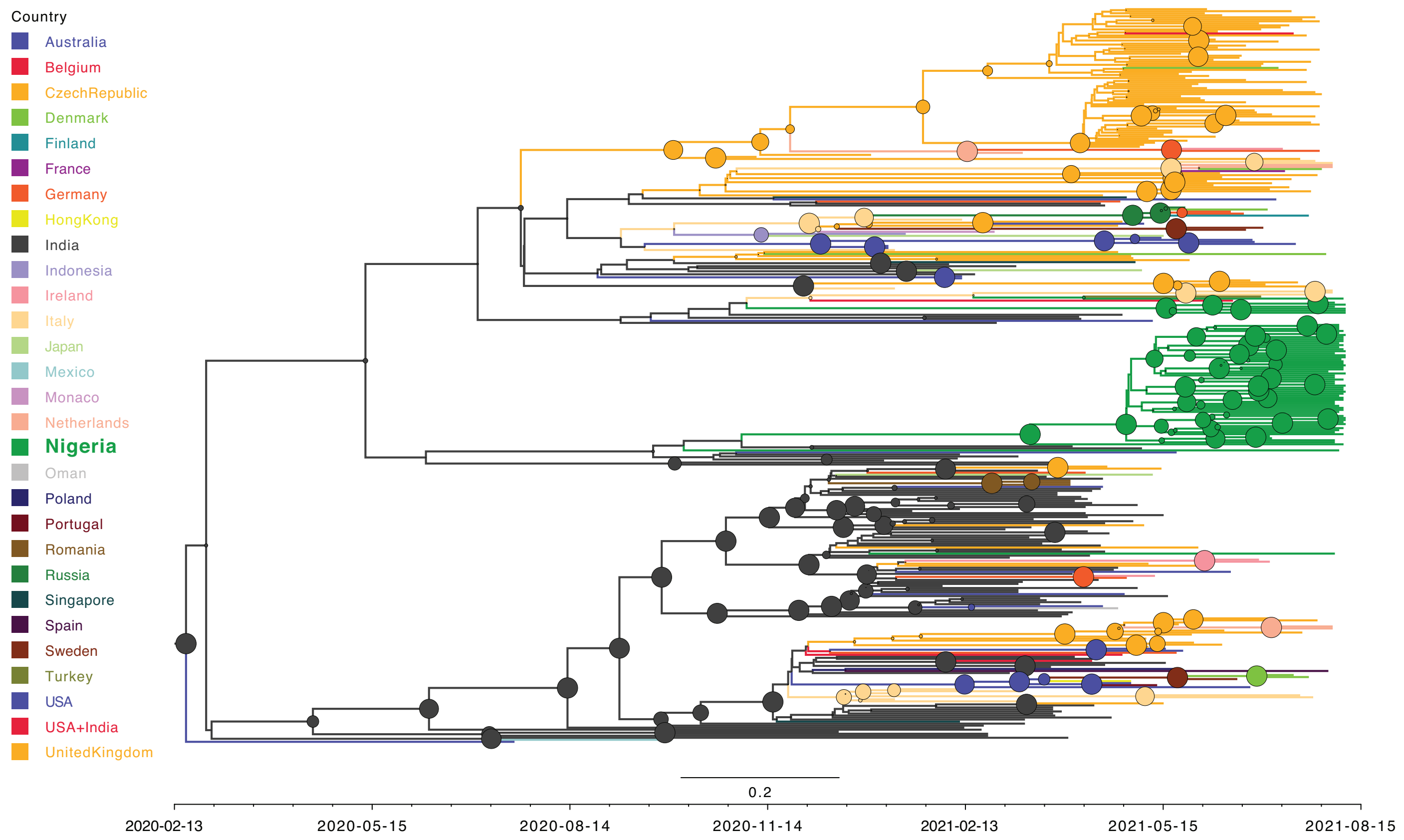

Supplementary Figure 5. Phylodynamic tree of Nigeria Delta sequences compared to global Delta sequences. Maximum clade credibility tree where branch colors represent the most probable geographical location of their descendent node inferred through Bayesian reconstruction of the ancestral state. All full genome Delta sequences from Nigeria and temporally and diversity subsampled Delta global sequences from GISAID as of August 3, 2021 were included in the analysis. The width of the node circles represents their posterior probability. Scale indicates number of substitutions per site and time.

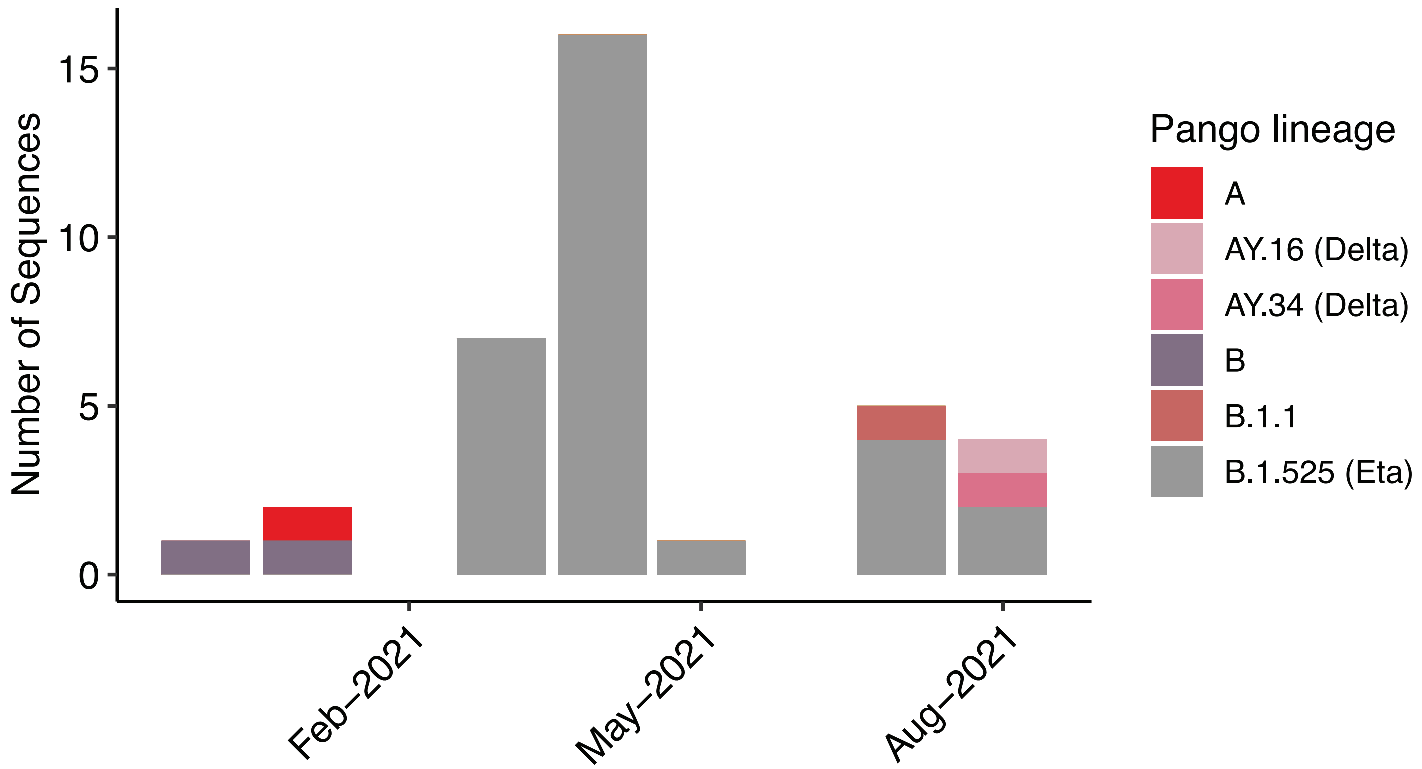

Supplementary Figure 6. Temporal SARS-CoV-2 lineage distribution in Mali. Distribution of the different clades per time found in Mali for this study.

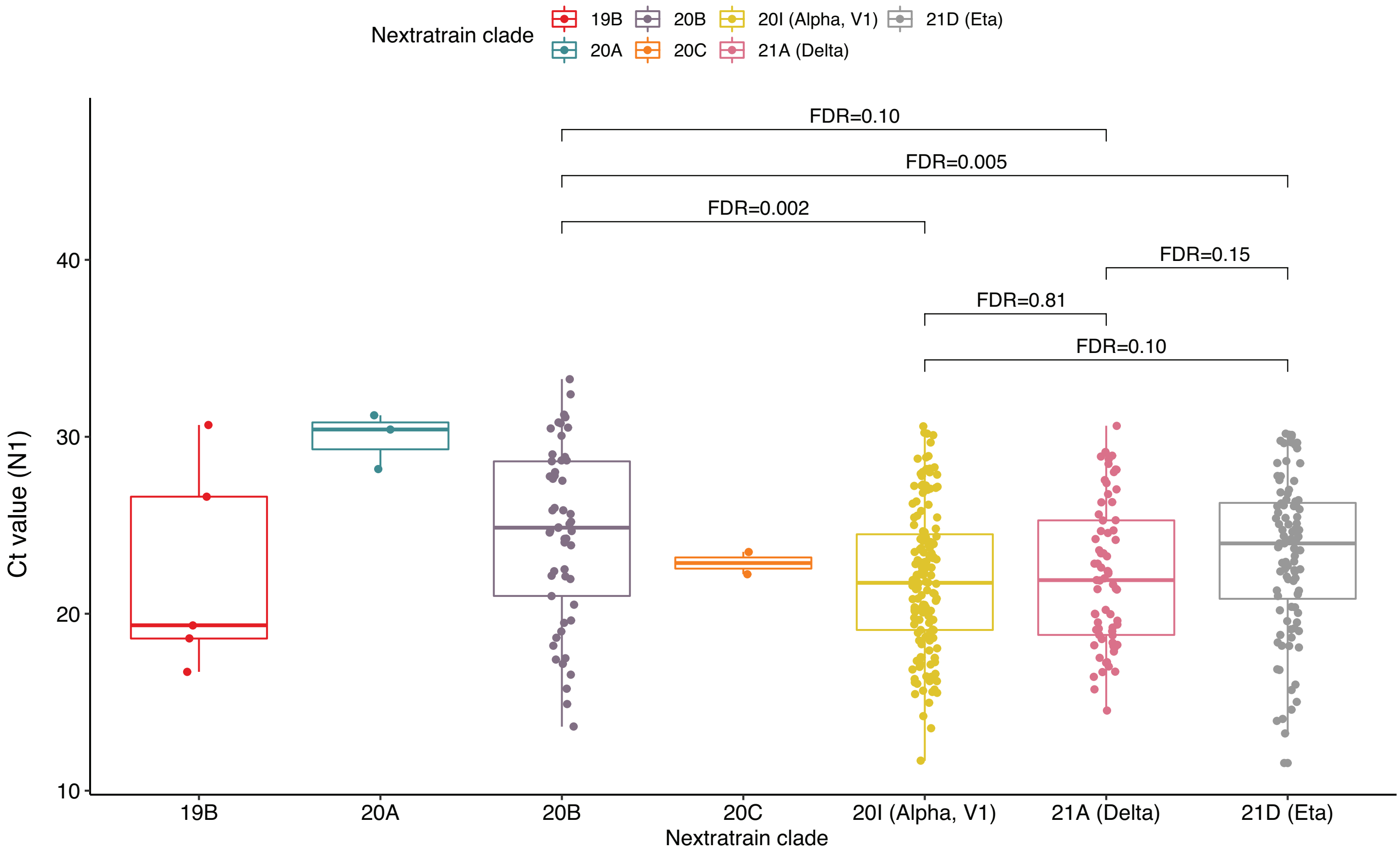

Supplementary Figure 7. PCR Cycle threshold (Ct) values of patient diagnostic samples grouped by pangolin lineage assignment. Ct values for the N1 probe set reported at the time of diagnosis were compared between lineages. A linear model was fitted to test for differences in Ct value between clades. Two-sided contrasts within the model performed between the majority clades are indicated. FDR was used to adjust for multiple comparisons. FDR values were considered statistically significant if less than 0.05. Tukey's box and whisker plots; box limits: interquartile range (IQR); middle line: median; vertical lines: data range (1st quartile − 1.5 IQR; 3rd quartile + 1.5 IQR). Dots represent all Ct values for each of the samples sequenced (n=378).

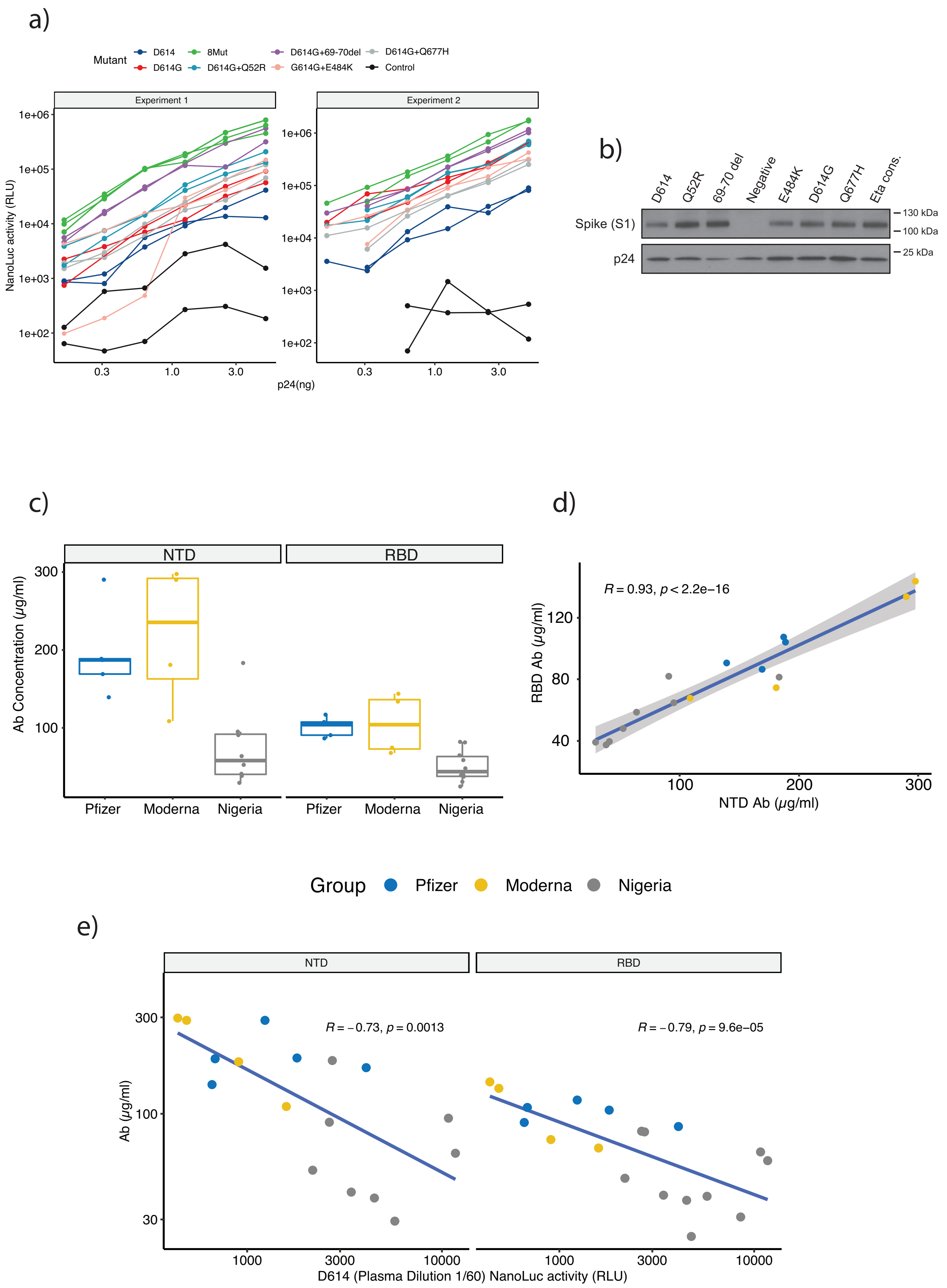

a)
Mutant
D614
D614G
8Mut
D614G+Q52R
D614G+69-70del
G614G+E484K
D614G+Q677H
Control
Experiment 1
Experiment 2
NanoLuc activity (RLU)
1e+06
1e+05
1e+04
1e+03
1e+02
0.3
1.0
3.0
p24(ng)
b)
D614
Q52R
69-70 del
Negative
E484K
D614G
Q677H
Eta cons.
Spike (S1)
p24
130 kDa
100 kDa
25 kDa
c)
NTD
RBD
Ab Concentration (μg/ml)
300
200
100
Pfizer
Moderna
Nigeria
d)
R = 0.93, p < 2.2e−16
RBD Ab (μg/ml)
120
80
40
100
200
300
NTD Ab (μg/ml)
Group
Pfizer
Moderna
Nigeria
e)
NTD
RBD
R = −0.73, p = 0.0013
R = −0.79, p = 9.6e−05
Ab (μg/ml)
300
100
30
1000
3000
10000
D614 (Plasma Dilution 1/60) NanoLuc activity (RLU)

Supplementary Figure 8. Cell entry of pseudotyped viruses with B.1.525 Spike mutations and Spike antibody concentrations in the tested sera. a) Nanoluc activity measured in relative light units (RLU) of the mutants tested, including D614G and D614. Lines colored by mutant represent replicates for each mutant in the two independent experiments performed. Dots are nanoluc activity values per reporter virus concentration. b) Western blot depicting the amount of the indicated Spike protein mutant incorporated into each preparation of virus-like particles. The HIV-1 capsid protein p24 is used as the loading control. The blot was run and probed two independent times; one representative image is shown. c) Spike NTD-binding and RBD-binding antibody concentrations calculated for each of the sera used in the neutralization experiments. Tukey's box and whisker plots; box limits: interquartile range (IQR); middle line: median; vertical lines: data range (1st quartile − 1.5 IQR; 3rd quartile + 1.5 IQR). Dots represent all values for each of the sera used per group (Pfizer [n=4], Moderna [n=4], and convalescent [n=9]). d) Correlation between RBD and NTD antibodies in each serum. e) Correlation between RBD and NTD antibodies and Nanoluc activity of D614 mutant at the lowest dilution used in the experiments (1:60). A very significant negative correlation between antibody concentration and reporter virus is found for both NTD and RBD indicating their effective neutralization of D614 mutant. For d and e dots are colored by the sera group, line represents linear regression and grey area is the regression confidence interval. Spearman rho and p-value for Spearman's rank correlation two-sided test are shown.

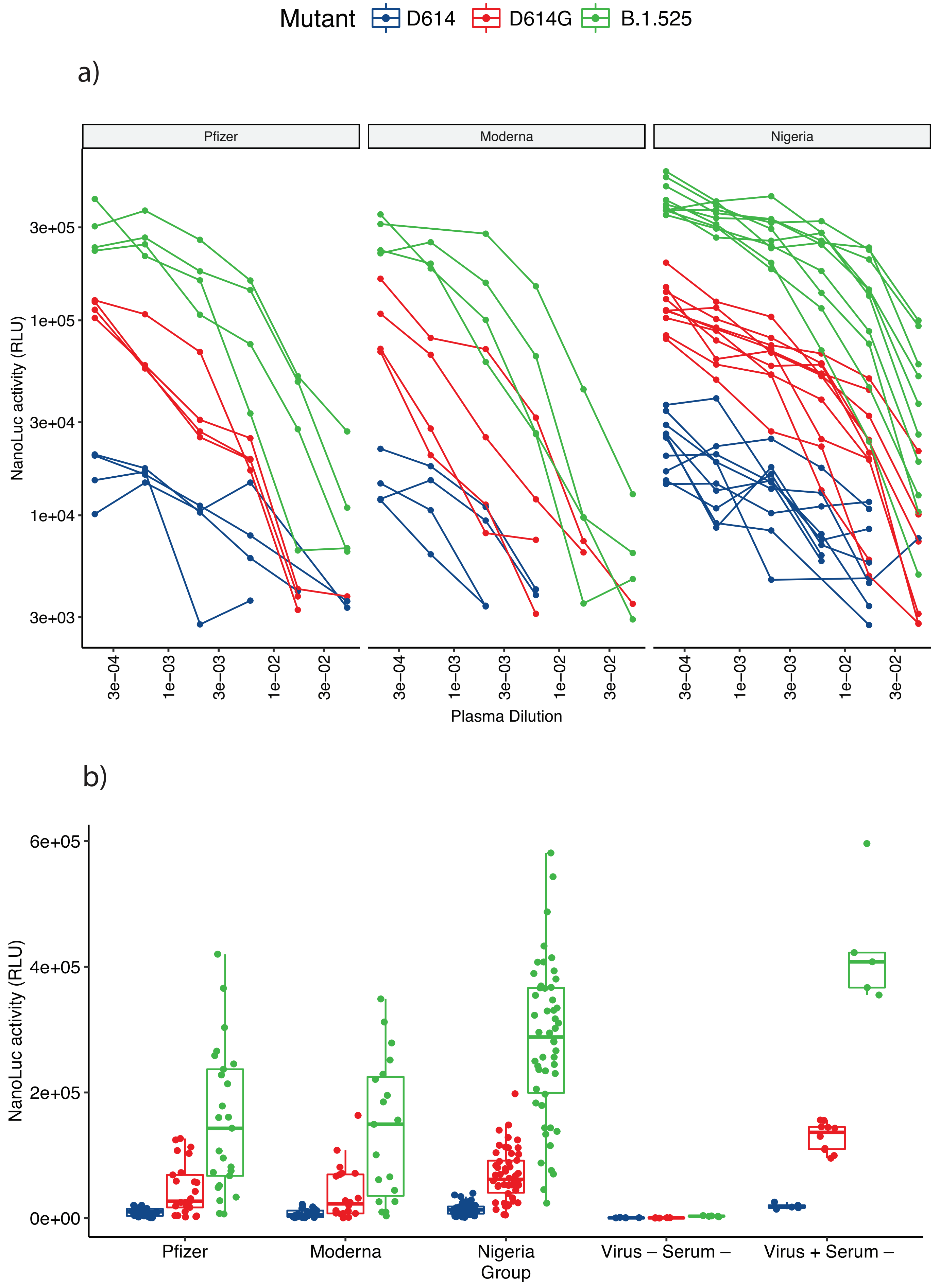
Mutant
D614
D614G
B.1.525
a)
Pfizer
Moderna
Nigeria
NanoLuc activity (RLU)
3e+05
1e+05
3e+04
1e+04
3e+03
3e−04
1e−03
3e−03
1e−02
3e−02
Plasma Dilution
b)
NanoLuc activity (RLU)
6e+05
4e+05
2e+05
0e+00
Pfizer
Moderna
Nigeria
Virus − Serum −
Virus + Serum −
Group

Supplementary Figure 9. Dilution curves for the different plasma samples used and all nanoluc activity values. a) Nanoluc activity values per plasma dilution. Lines represent each serum and dots are the nanoluc activity values per each serum dilution. Lines are colored by the mutant tested for every serum. b) Boxplot representing all nanoluc activity luminescence RLU values obtained in the neutralization experiments including the controls that were used to normalize these luminescence values for EC_{50} estimation. Boxes are colored by mutant and dots represent all luminescence values for each of the 5 dilutions performed with every mutant for all the sera per group (Pfizer [n=4], Moderna [n=4], and convalescent [n=9]). Positive controls included virus without serum (Virus+ Serum-) and negative controls did not include either virus or sera (Virus- Serum-). Tukey's box and whisker plots; box limits: interquartile range (IQR); middle line: median; vertical lines: data range (1st quartile − 1.5 IQR; 3rd quartile + 1.5 IQR).

Supplementary Table 1. Characteristics of the sera used for neutralization assays.

Post-Vaccination Sample ID	Vaccination Manufacturer	Date of 1st Dose	Date of 2nd Dose	Date of Blood Draw	Days Since Full Vaccination	Sex	Age Range
4	Pzifer	Dec-2020	Jan-2021	Feb-2021	14	M	36-40
5	Pzifer	Dec-2020	Jan-2021	Feb-2021	14	M	36-40
6	Pzifer	Dec-2020	Jan-2021	Feb-2021	14	M	21-25
13	Pfizer	Feb-2021	Mar-2021	Mar-2021	6	M	31-35
8	Moderna	Feb-2021	Mar-2021	Mar-2021	19	F	21-25
9	Moderna	Feb-2021	Mar-2021	Mar-2021	19	F	21-25
10	Moderna	Feb-2021	Mar-2021	Mar-2021	25	M	21-25
11	Moderna	Feb-2021	Mar-2021	Mar-2021	24	F	51-55
12	Moderna	Jan-2021	Feb-2021	Mar-2021	33	M	26-30
**Convalescence Sample ID**	**N CT Value**	**Date of Diagnosis**		**Date of Blood Draw**	**Days Since Diagnosis**	**Sex**	**Age Range**
28	18.79	May-2020		May-2020	5	F	31-35
29	28.32	May-2020		May-2020	5	M	46-50
30	26.12	May-2020		May-2020	5	F	31-35
33	21.82	May-2020		May-2020	5	M	46-50
35	26.06	May-2020		May-2020	5	M	46-50
39	22.08	May-2020		May-2020	5	M	31-35
42	26.54	May-2020		Jun-2020	36	F	31-35
66	23.44	Jun-2020		Jun-2020	10	M	21-25
68	26.19	Jun-2020		Jun-2020	5	F	26-30
69	27.21	Jun-2020		Jun-2020	5	F	26-30
