# Supplementary material for: Multiple expansions of globally uncommon SARS-CoV-2 lineages in Nigeria
Source: Nat Commun. 2022 Feb 3;13:688. doi: 10.1038/s41467-022-28317-5 (PMC8813984; doi:10.1038/s41467-022-28317-5)
Supplement: Supplementary file 2 — Description of Additional Supplementary Files [file 41467_2022_28317_MOESM2_ESM.pdf]

Description of Additional Supplementary Files

File Name: Supplementary Data 1
Description: Accession numbers, collection dates, and Pango.Lineage associated with the sequences obtained for the study.

File Name: Supplementary Data 2
Description: Accession numbers and collection dates for all GISAID sequences used in this study, and acknowledgement for authors, originating and submitting laboratories. All submitters may be contacted via https://www.gisaid.org/
