# Supplementary figures and images for: Multiple expansions of globally uncommon SARS-CoV-2 lineages in Nigeria
Source: Nat Commun. 2022 Feb 3;13:688. doi: 10.1038/s41467-022-28317-5 (PMC8813984; doi:10.1038/s41467-022-28317-5)

SOURCE DATA

SUPPLEMENTAL FIGURE 8b

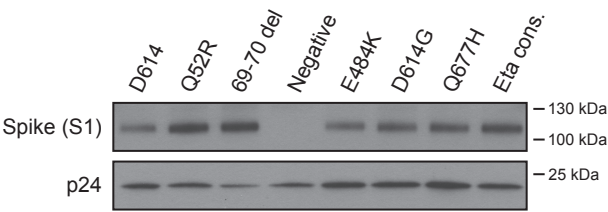

Full scan (anti-Spike S1)

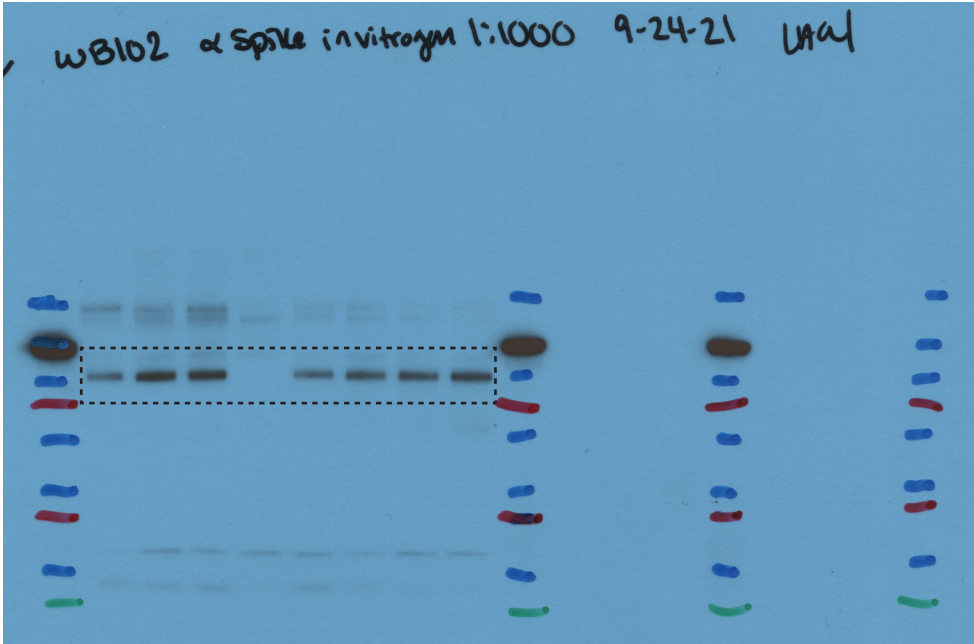

Full scan (anti-p24)

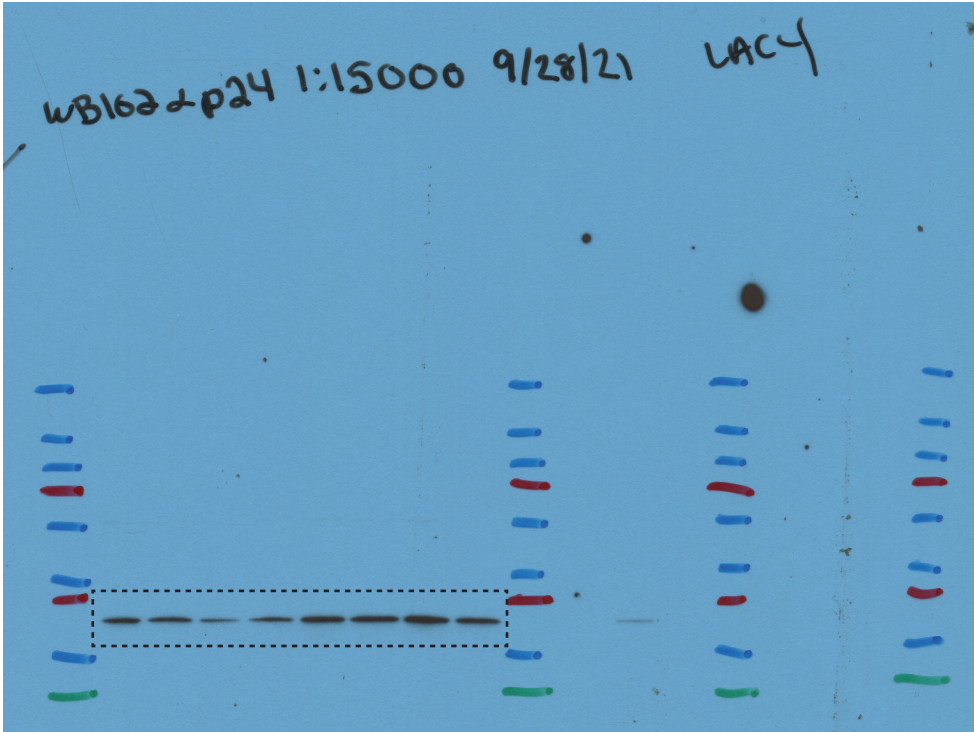

Supplement: Supplementary file 5 — Source Data [file 41467_2022_28317_MOESM5_ESM.pdf]
